# Multi‐Level Confinement Single‐Molecule Charge Transfer Activated PRET for Near‐Infrared Targeted Cell Imaging

**DOI:** 10.1002/advs.202522017

**Published:** 2026-02-19

**Authors:** Zhuo Lei, Sai Li, Pei‐Ao Sun, Yong Chen, Xuejian Zhang, Yu Liu

**Affiliations:** ^1^ College of Chemistry State Key Laboratory of Elemento‐Organic Chemistry Nankai University Tianjin P. R. China

**Keywords:** cell imaging, charge transfer, multi‐level confinement, near‐infrared delayed fluorescence emission, phosphorescent resonance energy transfer

## Abstract

This work reports an interesting phosphorescent resonance energy transfer (PRET) system with a large Stokes shift (320 nm) and near‐infrared (NIR) delayed fluorescence emission for high‐performance bioimaging, where a tailored charge transfer supramolecular assembly was constructed from dibenzyl‐bridged pyridinium derivative (G1), cucurbit[8]uril (CB[8]), and *β*‐cyclodextrin‐grafted hyaluronic acid (HACD). The rigid dibenzyl linker of G1 induces a unique zigzag conformation, enabling G1 to form an antiparallel charge transfer complex with CB[8], giving a binding constant as high as 5.49 × 10^6^ m ^−1^ and triggering a nanofiber‐to‐nanorod topological transformation that boosts energy transfer efficiency. Significantly, multi‐level confinement by CB[8] and HACD aligns G1 into donor–acceptor arrays, realizing single‐molecule PRET from bromophenyl pyridinium (phosphor donor) to anthracenyl pyridinium (acceptor). This unprecedented process expands 500 nm phosphorescence to 650 nm NIR delayed fluorescence with a lifetime recorded as 11.20 µs, overcoming traditional PRET's short‐wavelength limitation. Crucially, the system achieves successful NIR channel labeling/detection in living cells, demonstrating its bioimaging potential. This work advances supramolecular PRET design via rational structural tailoring and confinement engineering, holding broad significance for cell imaging, information encryption, and anti‐counterfeiting.

## Graphical abstract

The cascade supramolecular assembly of charge‐transfer arrays not only triggered a topological transformation from nanofiber to nanorod and vesicles, but also promoted single‐molecule phosphorescent resonance energy transfer, expanding phosphorescence emission at 500 nm to near‐infrared delayed fluorescence emission at 650 nm with a lifetime of 11.20 µs for tumor‐targeted mitochondrial imaging.

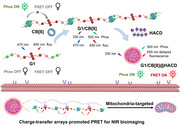

## 1 Introduction

Recently, near‐infrared (NIR) luminescence materials have widespread application in sensors [1, 2, 3], bioimaging [4, 5, 6, 7, 8, 9, 10], disease theranostics [11, 12], and photocatalytic biorthogonal chemistry [13, 14, 15] due to their excellent deep tissue penetrations, fast response speed, and low background interference. However, traditional NIR luminescence materials usually involve numerous challenges, such as burdensome chemical synthesis and modification of chromophore, low quantum efficiency, and thermal quenching. The capsulation of NIR chromophore by supramolecular macrocyclic hosts such as cyclodextrins [16, 17, 18], pillararenes [19, 20], and cucurbiturils [21, 22] can inhibit the formation of H‐aggregates and restrict the chromophore motions, which has been validated as an effective strategy to improve the photophysical properties of NIR luminescence materials. Furthermore, a fluorescent/phosphorescent resonance energy transfer (FRET/PRET)‐based artificial light‐harvesting system that transfers energy from the excited singlet/triplet state of emitters donor to the singlet state of chromophore acceptor, is considered a fascinating protocol to extend the emitters to the NIR region [23, 24, 25, 26, 27]. However, in addition to the overlap of spectra, the realization of FRET/PRET requires the spatial proximity of the donor/acceptor and suitable orientation of transition dipole moments [28]. In this regard, macrocyclic confinement and supramolecular cascade assembly not only facilitate the generation of phosphorescence by the restriction of molecular motion to suppress the energy loss from non‐radiative transition, but also bring the energy donor and acceptor closer together spatially and stabilize the singlet/triplet excitons, thereby enhancing the possibility of FRET/PRET. For example, Huang et al. reported a PRET system where the 4,4'‐biphenol, 1,1'‐bi‐2‐naphthol, and 1,8‐naphthalimide were employed as phosphorescence donors and doped with bodipy (BCA, BPH) or rhodamine derivatives (R123, RdB, and R101) as acceptors in a polyvinyl alcohol (PVA) matrix to form a series of organic afterglow materials with narrowband emission and high color purity via hydrogen‐bonding interaction [29]. Li et al. reported that bright and persistent red/NIR afterglow material was realized by highly efficient PRET processes leveraging branched RTP luminogens as energy donors and red/NIR dyes as energy acceptors. Owing to the highly branched luminogens enabling dyes encapsulation and confinement, the non‐radiative transition was suppressed, and the material performed high temperature resistance up to 413 K [30]. We also reported that a dodecyl‐chain‐bridged 6‐bromoisoquinoline derivative as phosphorescence donor achieved energy transfer to Nile blue or tetrakis(4‐sulfophenyl)porphyrin and NIR long‐lived emission with a high donor/acceptor ratio via the capsulation of CB[7] and the secondary confinement of HACD [31]. Although substantial progress has been made in constructing near‐infrared luminescent materials via FRET, most of these heterogeneous systems based on donor–acceptor doping suffer from drawbacks such as unfavorable distance for energy transfer, poor signal‐to‐noise ratio, and concerning stability. Single‐molecule fluorescence resonance energy transfer with a single electron‐donor/acceptor pair, whose transfer efficiency is directly determined by the intramolecular donor–acceptor distance and dipole orientation, exhibits sensitive and stable signals along with robust interference resistance, gaining wide popularity in the design of advanced NIR materials [32, 33]. Nevertheless, single‐molecule phosphorescent resonance energy transfer achieving NIR delayed fluorescent emission in a charge transfer complex by multi‐level macrocyclic confinement and tuning PRET process via charge transfer interaction is rarely reported, to the best of our knowledge.

**SCHEME 1:**
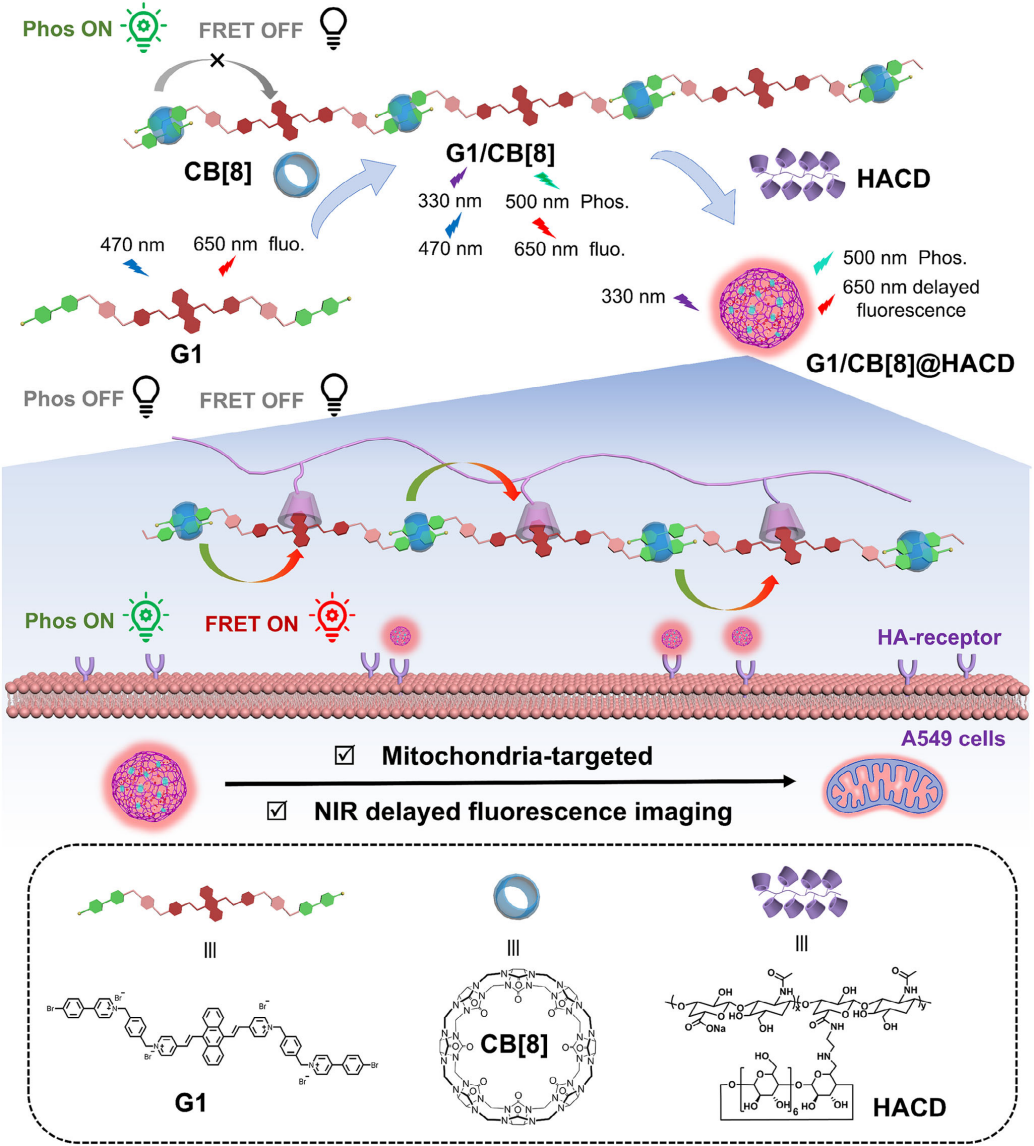
Schematic illustration of multi‐level macrocyclic confinement activated intramolecular phosphorescent resonance energy transfer for near‐infrared targeted cell imaging.

In this work, we wish to report an efficient single‐molecule PRET system based on multi‐level macrocyclic confinement, which was constructed by rigid dibenzyl‐bridged pyridinium derivative (G1), cucurbit[8]uril (CB[8]), and *β*‐cyclodextrin (*β*‐CD) modified hyaluronic acid (HACD), contributing to the targeted cancer cell imaging with a large Stokes shift of 320 nm and NIR delayed fluorescent emission (Scheme 1). Benefiting from the confinement effect of cucurbit[8]uril effectively promots the intersystem crossing process and inhibits the non‐radiative transition caused by the molecular vibration and quenchers, the binary assembly of CB[8] and G1 activated the bromophenyl pyridinium unit of distinct intense phosphorescence emission around 500 nm. After the further assembly with HACD via the confinement effect of *β*‐CD to anthracenyl group and electrostatic interaction with HA, an intramolecular PRET process derived from phenyl pyridinium unit to anthracenyl pyridinium portion was achieved, which was accompanied by the transformation in topological morphology from nanorods to vesicle with good cell permeability, ultimately giving an NIR delayed fluorescence at 650 nm with a lifetime recorded as 11.20 µs. Taking advantage of the tumor‐targeting properties of HACD, the ternary supramolecular assembly with a large Stokes shift and long‐lived NIR photoluminescence was successfully employed for mitochondria‐targeted imaging of cancer cells.

## 2 Results and Discussion

**FIGURE 1:**
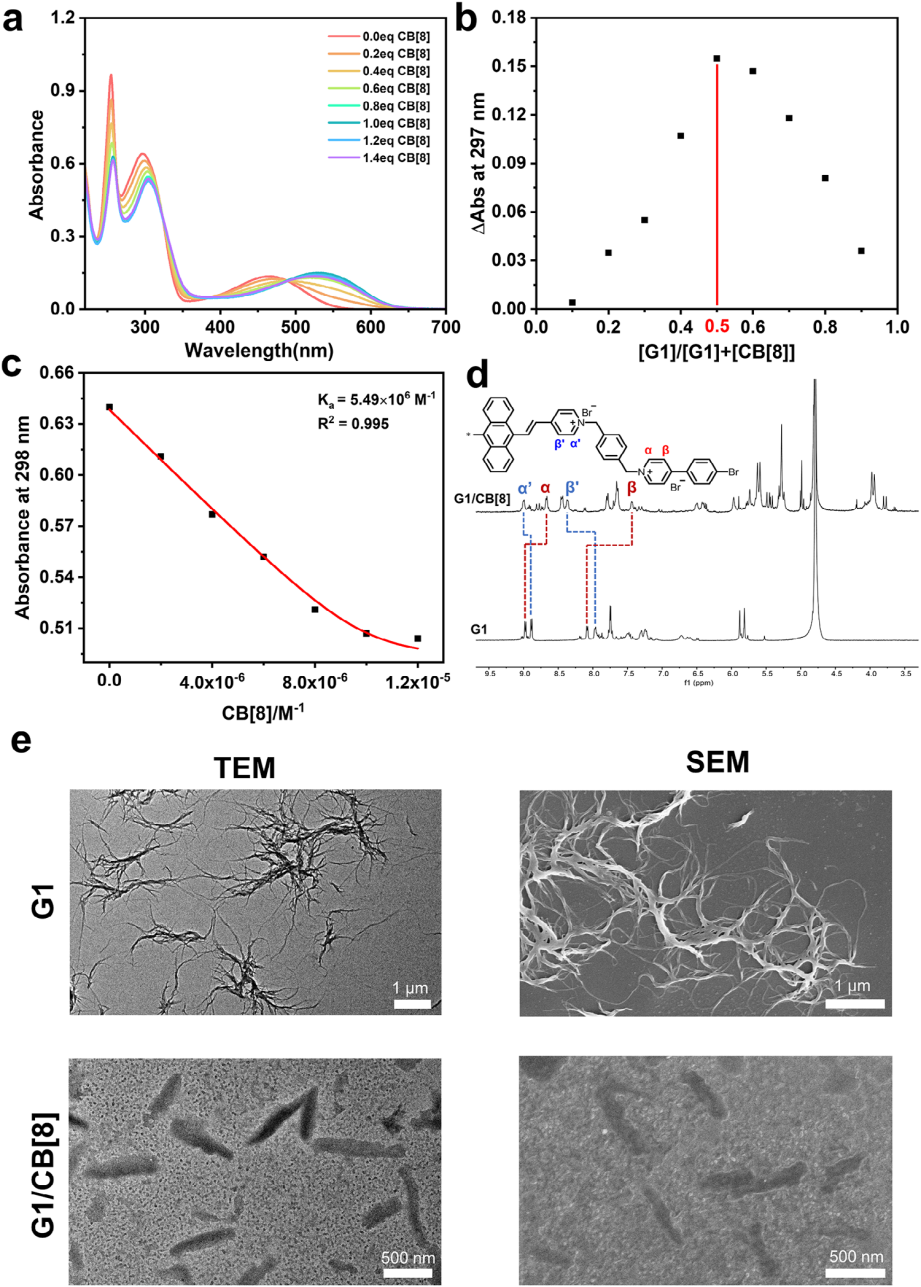
(a) UV–vis spectrum of the solution of **G1** (10 µm) in H_2_O with the addition of CB[8] (0–14 µm). (b) Job plot of **G1** with CB[8] obtained by UV–vis spectroscopy ([**G1**] + [CB[8]] = 20 µm). (c) Fitting curve for binding **G1** with CB[8] by a nonlinear least‐squares formula. (d) The ^1^H NMR spectra of **G1** and **G1**/CB[8]. (e) TEM and SEM images of **G1** and **G1**/CB[8].

**G1** was synthesized by a two‐step Mizoroki‐Heck reaction/alkyl substitution reaction and fully characterized through nuclear magnetic resonance (^1^H NMR, ^13^C NMR) and high‐resolution mass spectrometry (Figures S3–S5). First, UV–vis titration experiments were conducted to investigate the binding behavior between **G1** and CB[8]. With the addition of CB[8], the UV–vis spectra of **G1** exhibited a persistent redshift for its characteristic charge transfer absorption from 467 to 518 nm and stabilized after the addition of 1.0 equivalent CB[8] (Figure 1a), indicating that **G1** was encapsulated into the cavity of CB[8] at a 1:1 stoichiometry and the charge transfer interaction between anthracenyl as donor and pyridinium as acceptor was significantly enhanced due to the stabilization effect by CB[8]. The 1:1 stoichiometric binding ratio between **G1** and CB[8] was confirmed through Job's plot experiments (Figure 1b), and the binding constant was determined as 5.49 × 10^6^ m ^−1^ according to the UV–vis absorption titration fitted by a nonlinear least‐squares formula (Figure 1c). Furthermore, the ^1^H NMR and 2D NMR spectroscopy were carried out to infer the binding mode between **G1** and CB[8] (Figure 1d; Figures S8–S10). As shown in the ^1^H NMR spectrum of host‐guest complex (Figure 1d), the proton on anthracenyl pyridinium (α’ and β’) almost underwent significantly downshifting while the protons on bromophenyl pyridinium (α and β) shifted upfield, manifesting that the zigzag **G1** tend to form a terminal encapsulation of bromophenyl pyridinium units by CB[8] cavity with a head‐to‐tail binding mode. Meanwhile, the diffusion coefficient of guest molecule **G1** (D = 2.66 × 10^−10^ m/s^2^) was larger than the assembly **G1**/CB[8] (D = 9.37 × 10^−11^ m/s^2^) by an order of magnitude (Figure S10), which further verified the formation of n:n head‐to‐tail supramolecular pseudorotaxane for **G1**/CB[8]. Correspondingly, Transmission Electron Microscopy (TEM) and Scanning Electron Microscopy (SEM) were employed to reveal the change in topological structure after host‐guest complexation. As depicted in Figure 1e, the free guest molecule **G1** presented a spiral nanofiber with a length of approximately 3 µm, which might be attributed to the random self‐assembly driven by intermolecular charge transfer interaction. In comparison, the addition of CB[8] transformed the fibrous topological structure of **G1** into nanorods with varying lengths of 200–1000 nm, which was consistent with the ordered head‐to‐tail assembly mode for pseudorotaxane.

**FIGURE 2:**
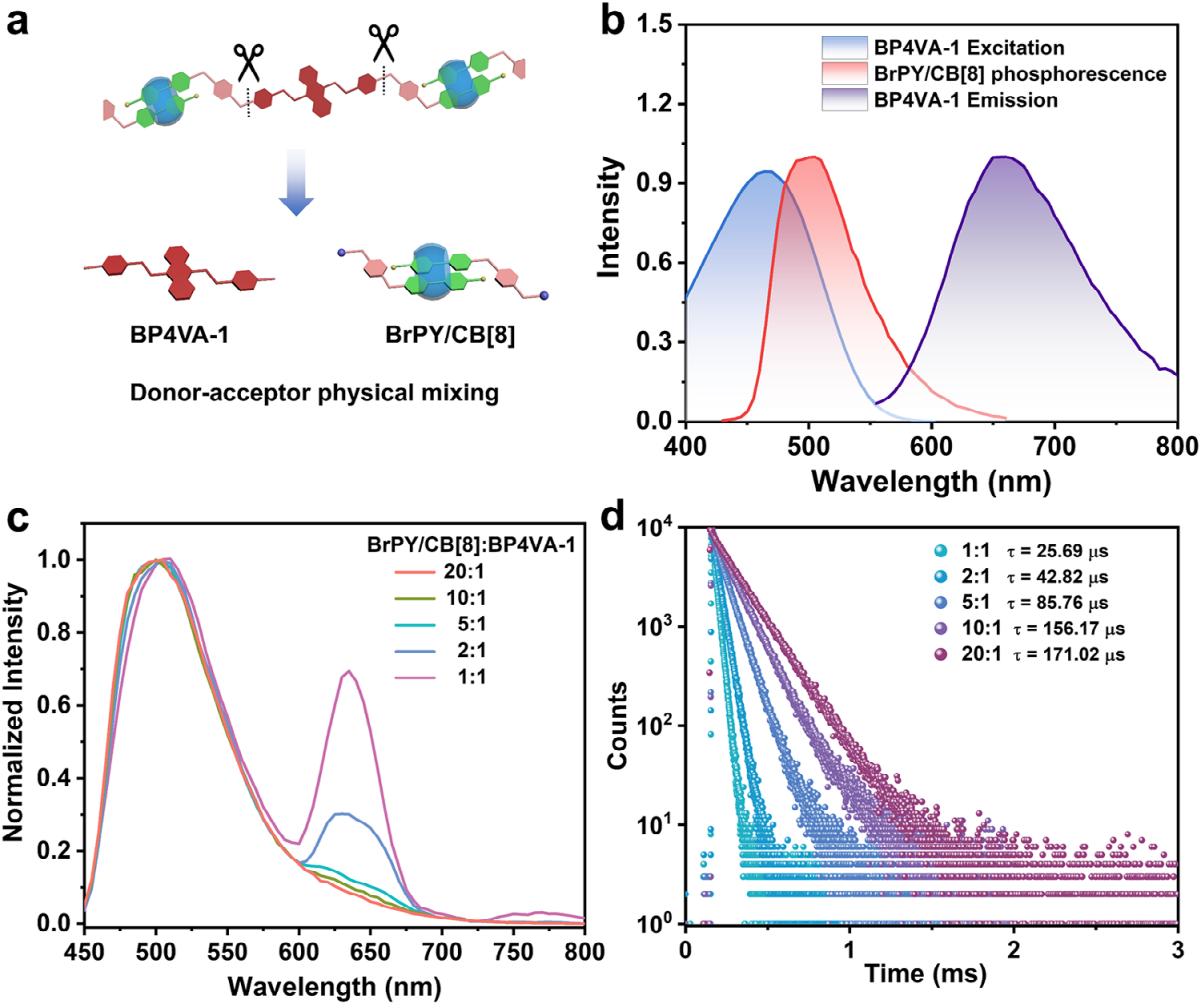
(a) Schematic illustration of donor–acceptor physical mixing. (b) The normalized phosphorescence emission spectrum of BrPY/CB[8] ([BrPY] = 25 µm, [CB[8]] = 12.5 µm), and the excitation and emission spectra of BP4VA‐1 ([BP4VA‐1] = 25 µm). (c) The normalized delayed spectra and (d) the time‐resolved decay curves of the doping system of BrPY/CB[8]‐BP4VA‐1@HACD ([BrPY] = 2[CB[8]] = 25 µm, [HACD] = 0.045 mg/mL) with different molar ratios of BP4VA‐1(delay time = 50 µs).

After fully characterizing the **G1** and **G1**/CB[8], the photoluminescence properties were explored. As shown in Figure S11, **G1** exhibited a fluorescence emission at 650 nm with an excitation of 470 nm, and no obvious phosphorescent signal was captured in the delayed spectrum. After adding 1.0 equivalent of CB[8], the fluorescence emission peak at 650 nm was enhanced, and there was an emission peak signal at 500 nm with an excitation of 330 nm in the delayed spectrum (Figure S12), corresponding to the confinement effect of CB[8] on bromophenyl pyridinium units and the supramolecular nanostructure formed by the linear rigid assembly layer by layer enabling valid shielding effect on the triplet quencher. As a result of the two components, bromophenyl pyridinium units and anthracenyl pyridinium units in **G1**, emerged phosphorescence emission at 500 nm and fluorescence emission at 650 nm under the excitation of 330 and 470 nm (Figure 2b), respectively, thereby satisfying the requirement of the substantial overlap of the spectra between donor and acceptor, we explored the construction of single‐molecule phosphorescent energy resonance transfer system. First, the reference compounds, BrPY and BP4VA‐1 were synthesized to investigate the phosphorescent behavior by simple physical mixing of energy donor and acceptor (Figure 2a). As a result of the promotion of the intersystem crossing process and the inhibition of the non‐radiative transition by CB[8]’s confinement effect on guests, the reference molecule BrPY displayed strong RTP emission around 500 nm with a lifetime of 372.26 µs after assembly with CB[8] (Figure S13). The steady‐state PL spectrum of BP4VA‐1 excited by 470 nm presented an emission peak at 650 nm with a nanosecond lifetime measured as 0.55 ns (Figure S14), revealing the pure fluorescence properties of the anthracenyl pyridinium unit. Upon increasing the donor/acceptor ratio from 20:1 to 1:1, the NIR delay fluorescence emission intensity of the acceptor at 650 nm was gradually enhanced (Figure 2c), while the phosphorescence lifetime at 500 nm decreased from 171.02 to 25.69 µs (Figure 2d), implying the PRET process between BrPY/CB[8] to BP4VA‐1. However, **G1**/CB[8] failed to perform delay fluorescence emission at 650 nm with the excitation of 330 nm (Figure S12B).

**FIGURE 3:**
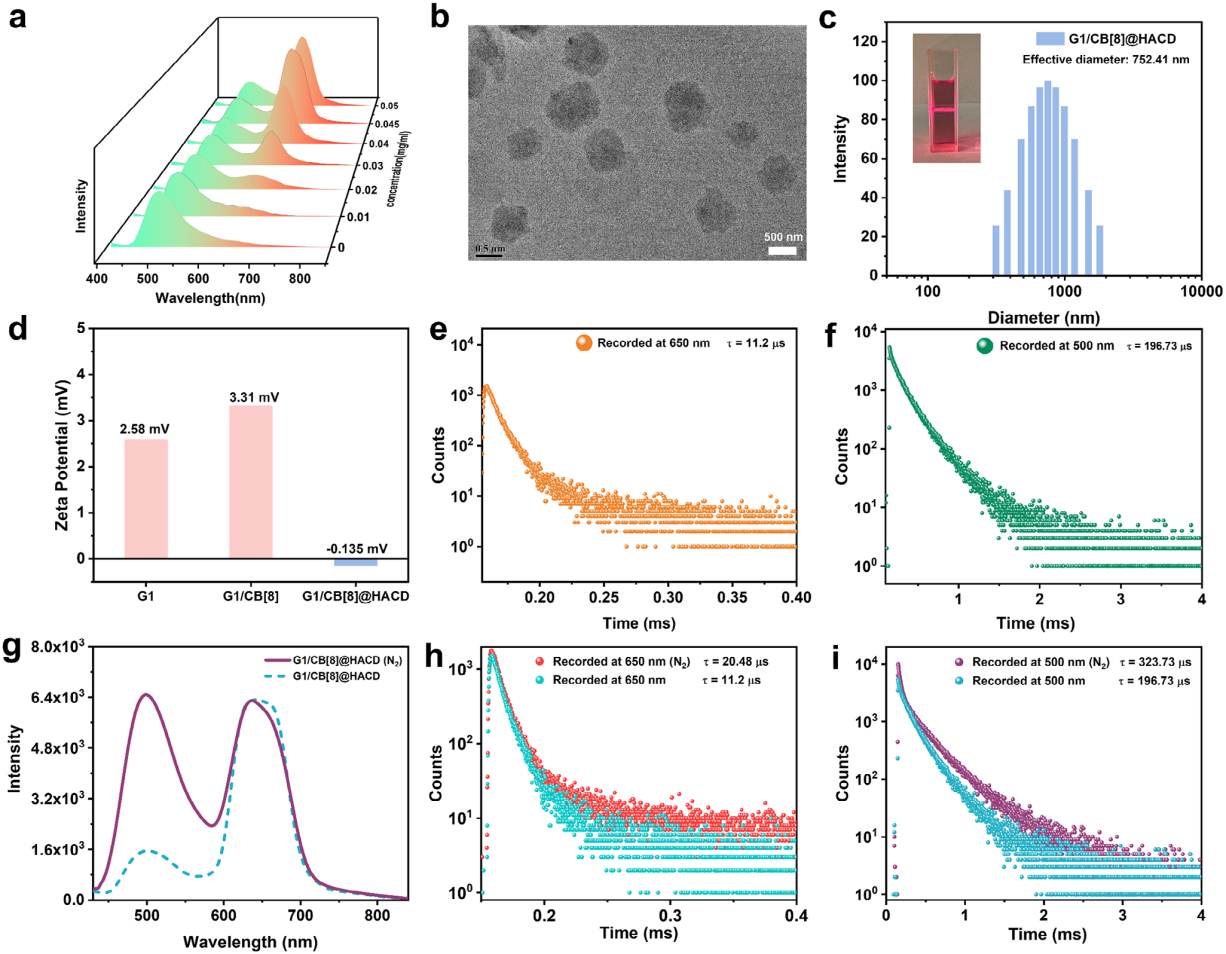
(a) The phosphorescence spectra of **G1**/CB[8] upon the addition of 0–0.05 mg/mL HACD ([**G1**] = 25 µm, [CB[8]] = 25 µm). (b) TEM image, (c) DLS profile, inset: the Tyndall effects in solution of **G1**/CB[8]@HACD, and (d) zeta potential of **G1**/CB[8]@HACD. The time‐resolved decay curves recorded at (e) 650 nm and (f) 500 nm of **G1**/CB[8]@HACD. (g) The delayed spectra and the time‐resolved decay curves recorded at (h) 650 nm and (i) 500 nm of **G1**/CB[8]@HACD under air or N_2_ atmosphere.

In order to achieve single‐molecule phosphorescent resonance energy transfer and improve biocompatibility for bioimaging, *β*‐CD‐grafted hyaluronic acid (HACD) was introduced to construct a ternary supramolecular assembly with **G1**/CB[8]. Taking advantage of the high negative charge density of HA and the capsulation of aryl groups by grafted *β*‐CD via the hydrophobic effect, we hypothesized that further restricting the host‐guest complexes after secondary assembly can inhibit non‐radiative transition and brought energy donor and acceptor close together in space, thereby enhancing the possibility of the occurrence of the PRET process. As expected, with the addition of HACD to the solution of **G1**/CB[8], the phosphorescent emission at 500 nm gradually decreased while a new emission peak at 650 nm in delayed spectra emerged, and stabilized at an amount of 0.045 mg/mL (Figure 3a). After determining the optimal amount of HACD (0.045 mg/mL), we explored the secondary assembly behavior and photophysical properties of **G1**/CB[8]@HACD. TEM image of **G1**/CB[8]@HACD revealed the exchanged topology from nanorods to vesicles (Figure 3b), and its size matched the average hydrodynamic diameter of 752 nm in dynamic light scattering (DLS) measurement (Figure 3c). Meanwhile, a significant Tyndall effect could also be observed in the **G1**/CB[8]@HACD solution (Figure 3c, inset), indicating the formation of supramolecular nano‐assemblies. Moreover, **G1** and **G1**/CB[8] possessed a positive zeta potential at +2.58 and +3.31 mV, which was converted to −0.135 mV with the addition of HACD (Figure 3d), revealing the successful construction of the ternary supramolecular assembly by electrostatic interaction. In order to verify the interaction between HACD and the anthracenyl group, BP4VA‐1 and *β*‐CD were selected as model compounds, and their binding behavior was investigated by ^1^H NMR spectra. As shown in Figure S15, the protons of BP4VA‐1 on anthracenyl (H_e_, H_f_) shifted downfield upon the addition of *β*‐CD, indicating the anthracenyl unit was encapsulated into the cavity of *β*‐CD. Furthermore, the addition of HACD effectively prolonged the fluorescence lifetime of BP4VA‐1, inferring that HACD could stabilize the singlet excitons of BP4VA‐1 (Figure S16), thereby further enhancing the possibility of PRET. Accordingly, the assembly of **G1**/CB[8]@HACD excited by 330 nm showed a phosphorescence emission at 500 nm with a lifetime of 196.73 µs, and a dominant emission band centered at 650 nm with a lifetime of 11.20 µs (Figure 3e,f), ascribing to the delayed fluorescence of the anthracenyl pyridinium unit by the PRET process. However, under the excitation of 450 nm, **G1**, **G1**/CB[8] and **G1**/CB[8]@HACD showed a fluorescence emission of 650 nm, where the lifetime was measured as 0.56, 0.63, and 0.77 ns (Figure S17), respectively, verifying that the HACD‐mediated NIR delayed fluorescence at 650 nm was realized by PRET process instead of direct excitation of energy acceptor. In addition, the lifetime of **G1**/CB[8]@HACD aqueous solution at 500 nm under nitrogen atmosphere was significantly increased from 196.73 to 323.73 µs with the phosphorescence emission intensity increased by four times, and the lifetime of the delayed fluorescence at 650 nm by PRET process was also increased from 11.2 to 20.48 µs (Figure 3g–i), due to the avoidance of the triplet electron quenching caused by oxygen.

Subsequently, Time‐Dependent Density Functional Theory (TD‐DFT) calculations were performed to further explore the mechanism of PRET in **G1**/CB[8]@HACD. The results revealed that the confinement effect of CB[8] stabilized the excited state of the coupled dimer, leading to a reduced energy gap between the highest occupied molecular orbital (HOMO) and the lowest unoccupied molecular orbital (LUMO) from 4.06 to 3.46 eV as well as a redshift of the low‐energy absorption band, which was consistent with the experimental spectral results (Figures S18–S21). It is noteworthy that replacing the negatively charged HACD with positively charged ε‐polylysine or neutral PEG in the assembly with **G1**/CB[8] failed to enhance the NIR emission at 650 nm (Figure S22), indicating that electrostatic interaction plays a crucial role in the actual structure of **G1**/CB[8]@HACD and facilitates single‐molecule PRET. Furthermore, the degradation of HACD by hyaluronidase can induce the disassembly of **G1**/CB[8]@HACD with the transformation of topological structure back from vesicles to nanorods (Figure S23), thereby achieving the dynamic regulation of single‐molecule PRET. As shown in Figure S24, the 650 nm delayed fluorescence signal of **G1**/CB[8]@HACD decreased markedly as the co‐incubation time with hyaluronidase increased. Meanwhile, the phosphorescence emission at 500 nm exhibited a characteristic trend of initial recovery and subsequent decline, which may be attributed to the fact that the preliminary degradation of **G1**/CB[8]@HACD by hyaluronidase resulted in the release of the dye molecules from the supramolecular assemblies, which abrogated the PRET process and consequently restored the phosphorescence signal. However, the further enzymatic degradation of HACD relieved the secondary confinement effect for **G1**/CB[8], leading to the subsequent decrease in phosphorescence intensity. Compared to commercial NIR dyes and state‐of‐the‐art PRET systems for NIR bioimaging, **G1**/CB[8]@HACD exhibited considerable quantum yields, long lifetime of NIR luminescence, and excellent photostability (Figures S25 and S26, Table S1), rendering it a promising candidate for near‐infrared delayed fluorescence imaging.

**FIGURE 4:**
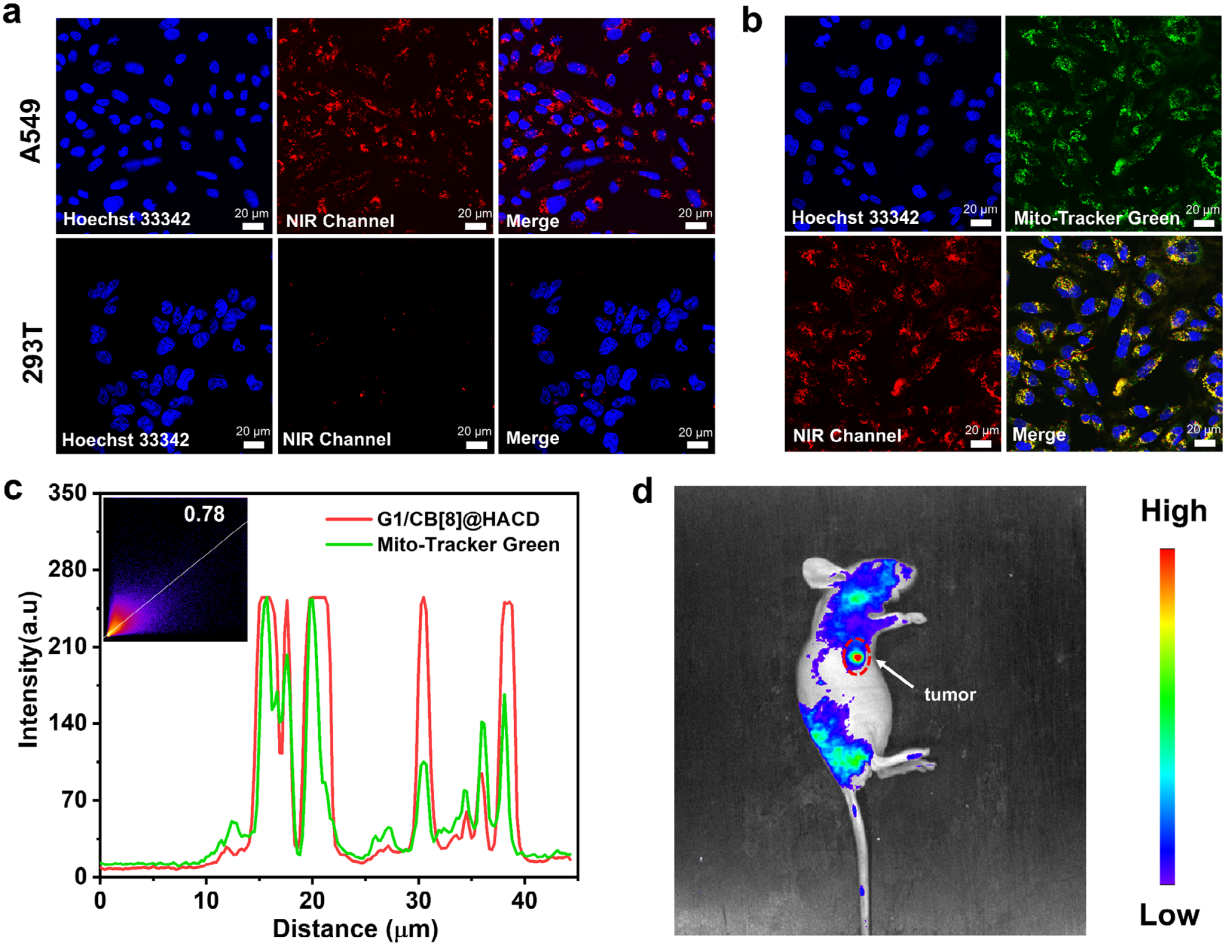
(a) The Confocal microscopy images of A549 and 293T cells in the presence of **G1**/CB[8]@HACD (10 µm). (b) The Confocal microscopy images of A549 incubated with **G1**/CB[8]@HACD (10 µm) and Mito‐Tracker Green (100 nm). (c) The Z‐stack image of the colocalization of **G1**/CB[8]@HACD and Mito‐Tracker Green. (d) In vivo fluorescence images of a mouse treated with **G1**/CB[8]@HACD.

Considering that the ternary nano‐supramolecular assembly **G1**/CB[8]@HACD constructed by PRET possessed good NIR delayed fluorescence emission properties and could be recognized by the overexpressed HA‐receptors on tumor cells, we explored its application in tumor‐targeted bioimaging. Human lung cancer cells (A549 cells) and human embryonic kidney cells (293T cells) were treated with **G1**/CB[8]@HACD for 12 h, respectively, and then incubated with Hoechst and Mito‐Tracker Green or Lyso‐Tracker Green for localization experiments. The distribution of **G1**/CB[8]@HACD in the organelles was observed by confocal laser scanning microscopy (CLSM). As shown in Figure 4a, A549 cells exhibited a bright NIR emission corresponding to the delayed fluorescence of **G1**/CB[8]@HACD, whereas almost no signal was found in the NIR channel for normal 293T cells, suggesting that **G1**/CB[8]@HACD was preferentially internalized into the cancer cells rather than normal cells by HA receptor‐mediated endocytosis. Furthermore, the NIR luminescence signal overlapped well with the signal of Mito‐Tracker Green, and the Pearson correlation coefficient was accordingly calculated as 78% (Figure 4b,c), revealing the ability of **G1**/CB[8]@HACD for targeted mitochondrial imaging in cancer cells. In addition, cytotoxicity was evaluated by CCK‐8 assay kit and the results indicated that the cell viability remained above 90% for both A549 cells and 293T cells after incubation for 24 h (Figure S27), demonstrating the excellent biocompatibility of **G1**/CB[8]@HACD. Encouraged by the outstanding mitochondrial‐targeted NIR imaging ability of **G1**/CB[8]@HACD in cellular level, in vivo imaging was conducted to further evaluate its potential for clinical translation. The animal usage and experimental procedures were approved by the Animal Experimentation Ethics Committee of Nankai University (2024‐SYDWLL‐000297), and all experimental protocols and operation procedures were performed in accordance with relevant guidelines. **G1**/CB[8]@HACD (100 µm, 0.13 mL) was administered to the A549‐bearing BALB/c‐nude mouse by tail vein injection, and its distribution was evaluated by in vivo imaging system (IVIS) after incubation for 12 h. As depicted in Figure 4d, **G1**/CB[8]@HACD was concentrated in the tumor tissue, which may be attributed to the enhanced permeability and retention effect (EPR) arising from its suitable size as well as its recognition by the abundant HA receptors on the tumor surface.

## 3 Conclusion

In summary, we have successfully constructed a single‐molecule PRET system through the macrocyclic confinement of CB[8] and the secondary assembly of HACD to achieve NIR delayed fluorescence emission for targeted cell imaging. The rigid dibenzyl linker of **G1** spanning between donor and acceptor in the PRET system allowed antiparallel stacking mode with CB[8] to form intermolecular charge transfer arrays, which realized the topological transformation of **G1** from nanofiber to nanorods, and then vesicles with stepwise addition of CB[8] and HACD. Meanwhile, the unique self‐assembly mode of ternary supramolecular assembly **G1**/CB[8]@HACD promoted the single‐molecule PRET from the bromophenyl pyridinium to anthracenyl pyridinium through multi‐level confinement effect, converting the phosphorescence emission at 500 nm to the NIR delayed fluorescence emission at 650 nm with a lifetime of 11.20 µs. Furthermore, this multi‐level confinement activated single‐molecule PRET system displayed a large Stokes shift of 320 nm and was successfully applied in mitochondria‐targeted tumor cell imaging with negligible cytotoxicity, which provides a convenient path for the construction single‐molecule PRET system and has potent to the development of the functional NIR luminescent materials.

## Conflicts of Interest

The authors declare no conflicts of interest.

## Supporting information




**Supporting File**: advs74513‐sup‐0001‐SuppMat.docx

## Data Availability

The data that support the findings of this study are available in the supplementary material of this article.
