# Supplementary material for: Multi‐Level Confinement Single‐Molecule Charge Transfer Activated PRET for Near‐Infrared Targeted Cell Imaging
Source: Adv Sci (Weinh). 2026 Feb 19;13(25):e22017. doi: 10.1002/advs.202522017 (PMC13137828; doi:10.1002/advs.202522017)
Supplement: Supplementary file 1 — Supporting File: advs74513‐sup‐0001‐SuppMat.docx [file ADVS-13-e22017-s001.docx]

**Multi-Level Confinement Single-Molecule Charge Transfer** **Activated PRET for Near-Infrared Targeted Cell Imaging**

*Zhuo Lei^+^, Sai Li^+^, Pei-Ao Sun, Yong Chen, Xuejian Zhang^*^, and Yu Liu^*^*

Z. Lei, S. Li, P. A. Sun, Y. Chen, X. Zhang, Y. Liu

College of Chemistry, State Key Laboratory of Elemento-Organic Chemistry, Nankai University, Tianjin 300071, P. R. China
E-mail: yuliu@nankai.edu.cn; 9820230017@nankai.edu.cn

[^+^] These authors contributed equally to this work.

1. **Materials and Instruments**

Unless additional stated, all reagents and solvents were available from commercial sources and used directly without any purification. ^1^H NMR and ^13^C NMR spectra were recorded through Bruker AV400 in the indicated solvents at 25 °C. Chemical shifts were referenced to the residual solvent peaks. DOSY spectra were measured on Bruker AVANCE III HD 400 spectrometer. High-resolution mass spectrometry (HR-MS) was recorded on a Q-TOF LC-MS in an Electrospray ionization (ESI) source. UV-vis data were collected on Shimadzu UV-3600 spectrophotometer. Photoluminescence (PL) spectrum and time-correlated decay profiles were documented on Edinburgh Instruments F900. The Transmission Electron Microscope (TEM) experiments were carried out on FEI Tecnai G2 F20 under 200 KV. Field emission scanning electron microscope (FE-SEM) were recorded on FEI Apreo S LoVac working at an accelerating voltage of 200 eV~30 keV. Dynamic light scattering (DLS) investigations were recorded with a DynaPro NanoStar dynamic light scattering detector. The Zeta potentials were examined on Brookhaven ZETAPALS/BI-200SM at 298 K. Laser scanning confocal microscopy (LSCM) were carried on Olympus FV1000. In vivo imaging experiments were conducted on Caliper Life Science IVIS Lumina II.

1. **Animals and Ethical statement**

BALB/c nude mice for in vivo imaging were purchased from Beijing FHK bioscience Co. Ltd. All the animals were acclimated under standard laboratory conditions including ventilated room, suitable temperature (25 ± 1 ℃), controlled humidity (60 ± 5 %) and a 12 h light/dark cycle. All procedures were conducted in accordance with the “Guiding Principles in the Care and Use of Animals” (China) and also assessed by the Animal Experimentation Ethics Committee of Nankai University, and the assigned approval number 2024-SYDWLL-000297.

1. **Compounds synthesis and characterization**

**Scheme S1.** Synthesis of **G1** and its reference molecules **BP4VA-1** and **BrPY**.

**BrPY**

**72.2%**

**Synthesis of** **BP4VA.** Compound **BP4VA** was synthesized according to the literature^1,2^. 4-Vinylpyridine (1.5 g, 14.27 mmol) and 9,10-dibromoanthracene (2.0 g, 5.95 mmol) were dissolved in 50 mL of dry DMF. Subsequently, K_2_CO_3_ (3.0 g, 21.7 mmol) and PdCl_2_ (50.0 mg) were added and the mixture was refluxed under N_2_ atmosphere for 12 h. The mixture was poured into 100 mL of water, filtered and subjected to column chromatography on silica gel (CH_2_Cl_2_/MeOH = 3%) to afford **BP4VA** (1.15 g, 50%). ^1^H NMR (400 MHz, CDCl_3_): δ 8.71 − 8.69 (m, 4H), 8.33 (dd, *J* = 6.8, 3.3 Hz, 4H), 8.18 (d, *J* = 16.55 Hz, 2H), 7.58 − 7.54(m, 4H), 7.53 (dd, *J* = 6.9, 3.2 Hz, 4H), 6.91 (d, *J* = 16.5 Hz, 2H).

**Synthesis of BrPY.** The acetonitrile solution (15 mL) containing 4-(4-bromophenyl) pyridine (585.3 mg, 2.5 mmol) was added dropwise to the acetonitrile solution (50 mL) of 1,4-dibromomethylbenzene (6.6 g, 25 mmol) and the reaction mixture was heated to 80 ℃ for 12 hours. After evaporating to remove solvent from the mixture, the residue was washed three times with ethyl acetate and dried to afford **BrPY** as a white powder (0.90 g, 72%). ^1^H NMR (400 MHz, DMSO-*d_6_*): δ 9.23 (d, J = 5.9 Hz, 2H), 8.54 (d, J = 6.6 Hz, 2H), 8.04 − 7.99 (m, 2H), 7.89 − 7.84 (m, 2H), 7.54 (s, 4H), 5.83 (s, 2H), 4.71 (s, 2H).

**Synthesis of G1****.** **BP4VA** (77 mg, 0.2 mmol) and **BrPY** (249 mg, 0.5 mmol) were dissolved in DMF (5 ml) and the reaction mixture was heated to 100 ℃ for 24 hours. After being cooled to room temperature, the mixture was filtered, washed with cold acetonitrile and ethyl acetate and dried to yield **G1** as a red powder (94 mg, 70.2%).^1^H NMR (400 MHz, DMSO-*d_6_*): δ 9.26 (dd, *J* = 13.7, 6.5 Hz, 8H), 8.98 (d, *J* = 16.5 Hz, 2H), 8.65 − 8.48 (m, 8H), 8.41 (dd, *J* = 6.8, 3.3 Hz, 4H), 8.11 − 7.93 (m, 4H), 7.95 − 7.78 (m, 4H), 7.70 (d, *J* = 1.6 Hz, 8H), 7.64 (dd, *J* = 6.6, 3.2 Hz, 4H), 7.30 (d, *J* = 16.4 Hz, 2H), 5.89 (d, *J* = 6.3 Hz, 8H). ^13^C NMR (101 MHz, DMSO-*d_6_*): δ 154.51, 152.96, 145.43, 145.09, 136.28, 135.93, 133.15, 133.13, 132.39, 130.74, 130.72, 30.11, 129.97, 129.78, 129.20, 127.09, 126.87, 126.58, 125.58, 125.48, 62.33. HRMS (ESI): m/z for C_66_H_52_Br_2_N_4_ calcd. [M-4Br]^4+^: 265.06291, found: 265.06330.

**Synthesis of BP4VA-1**. Compound **BP4VA** (96 mg, 0.25 mmol) and CH_3_I (142 mg, 1 mmol) were dissolved into DMF (5 mL) and the reaction mixture was heated to 85 ℃ for 24 hours. After being cooled to room temperature, the mixture was filtered, washed with cold acetonitrile and ethyl acetate, and dried to afford **BP4VA-1** as a red powder (87 mg, 52%). ^1^H NMR (400 MHz, DMSO-*d_6_*): δ 9.07 − 8.92 (m, 6H), 8.53 (d, *J* = 6.4 Hz, 4H), 8.44 (dd, *J* = 6.7, 3.3 Hz, 4H), 7.68 (dd, *J* = 6.8, 3.3 Hz, 4H), 7.32 (d, *J* = 16.5 Hz, 2H), 4.36 (s, 6H). ^13^C NMR (101 MHz, DMSO-*d_6_*): δ 152.08, 145.82, 138.01, 133.21, 132.33, 129.22, 127.10, 126.56, 124.79, 47.69.

**Figure S1.** ^1^H NMR spectrum of **BP4VA** (400 MHz, CDCl_3_, 298 K).

**Figure S2.** ^1^H NMR spectrum of **BrPY** (400 MHz, DMSO-*d_6_*, 298 K).

**Figure S3.** ^1^H NMR spectrum of **G1** (400 MHz, DMSO-*d_6_*, 298 K).

**Figure S4.** ^13^C NMR spectrum of **G1** (101 MHz, DMSO-*d_6_*, 298 K).


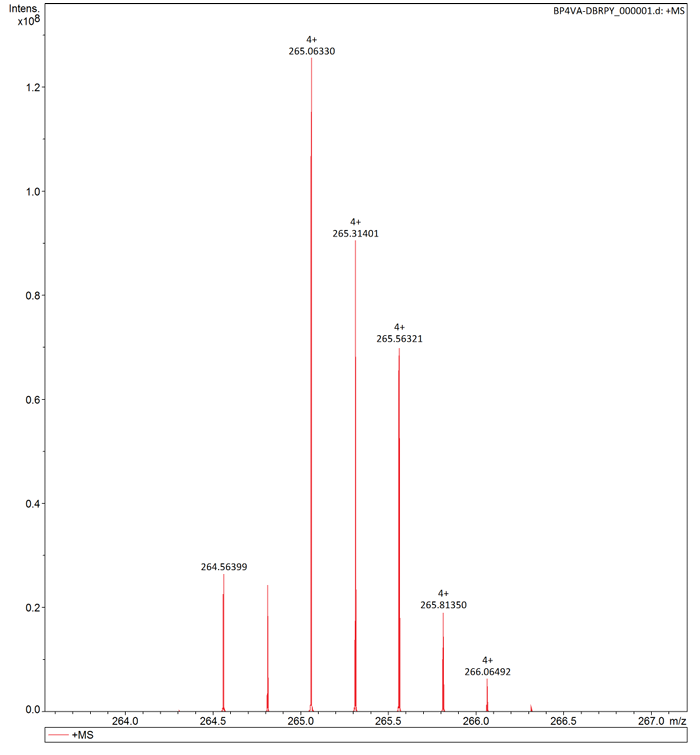


**Figure S5.** HR-MS spectrum of **G1**.

**Figure S6.** ^1^H NMR spectrum of **BP4VA-1** (400 MHz, DMSO-*d_6_*, 298 K).

**Figure S7.** ^13^C NMR spectrum of **BP4VA-1** (101 MHz, DMSO-*d_6_*, 298 K).


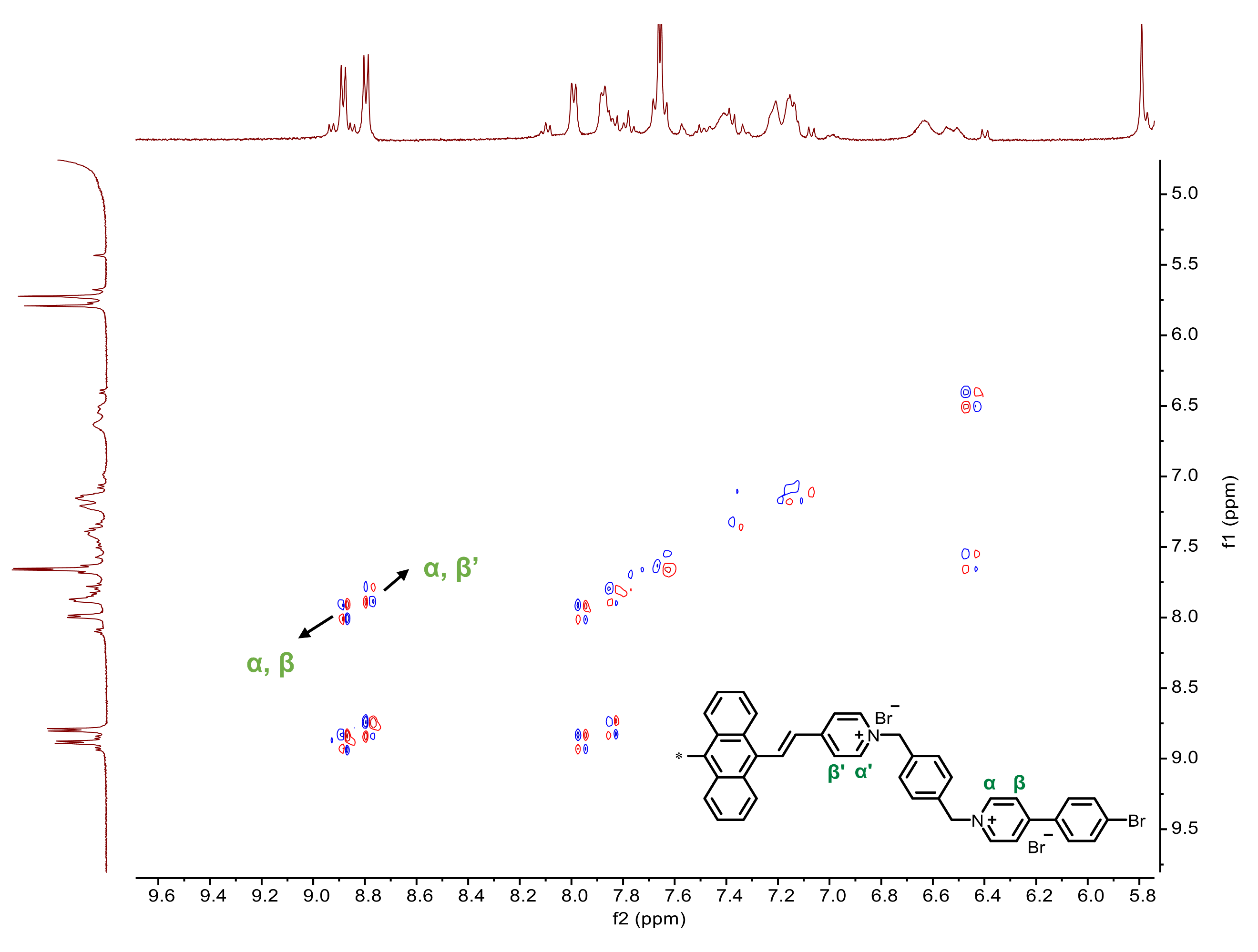


**Figure S8.** ^1^H-^1^H COSY spectrum of **G1** (400 MHz, D_2_O, 298 K).


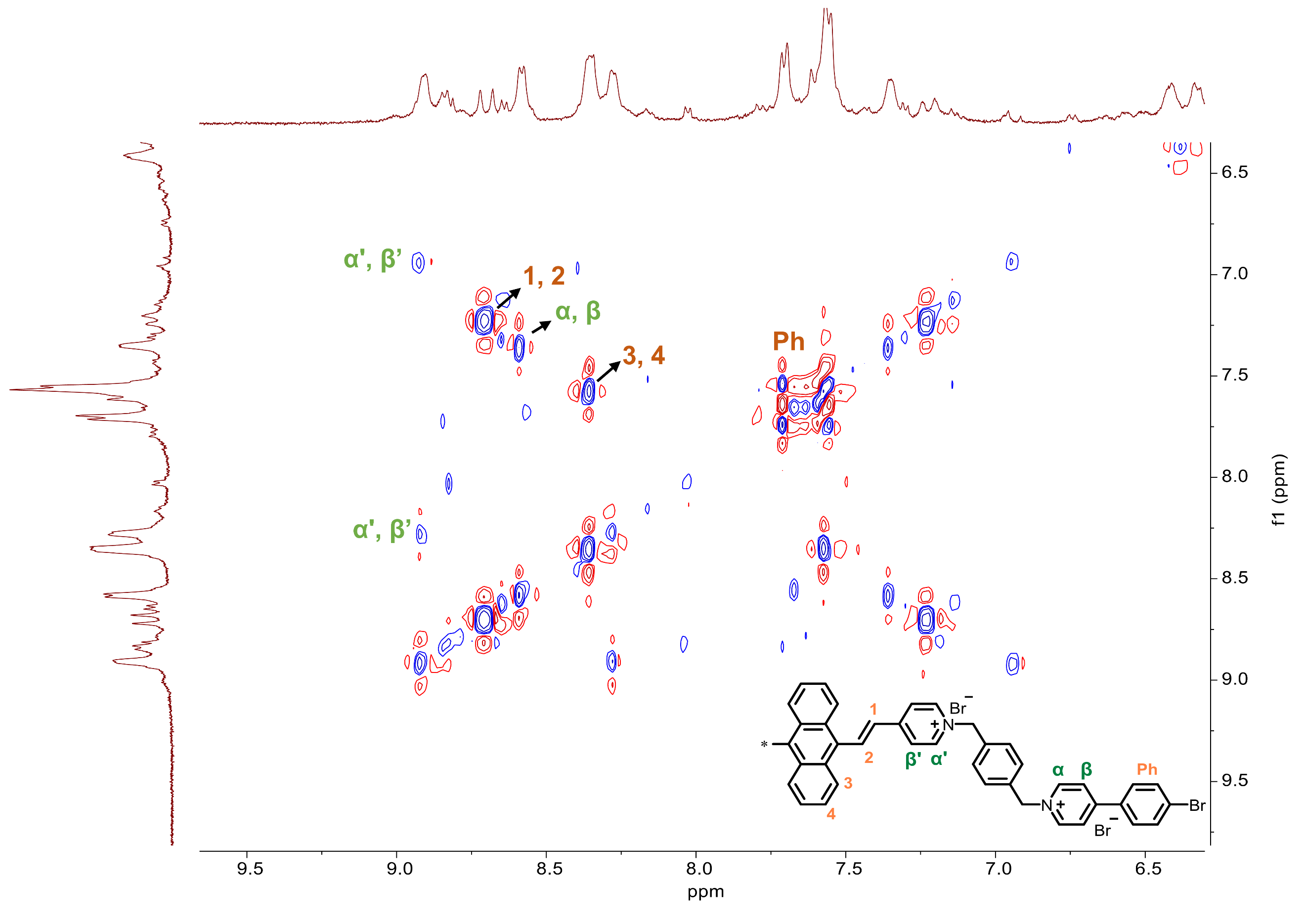


**Figure S9.** ^1^H-^1^H COSY spectrum of **G1/CB[8]** (400 MHz, D_2_O, 298 K). For clarity, the CB[8] is omitted for better visualization.


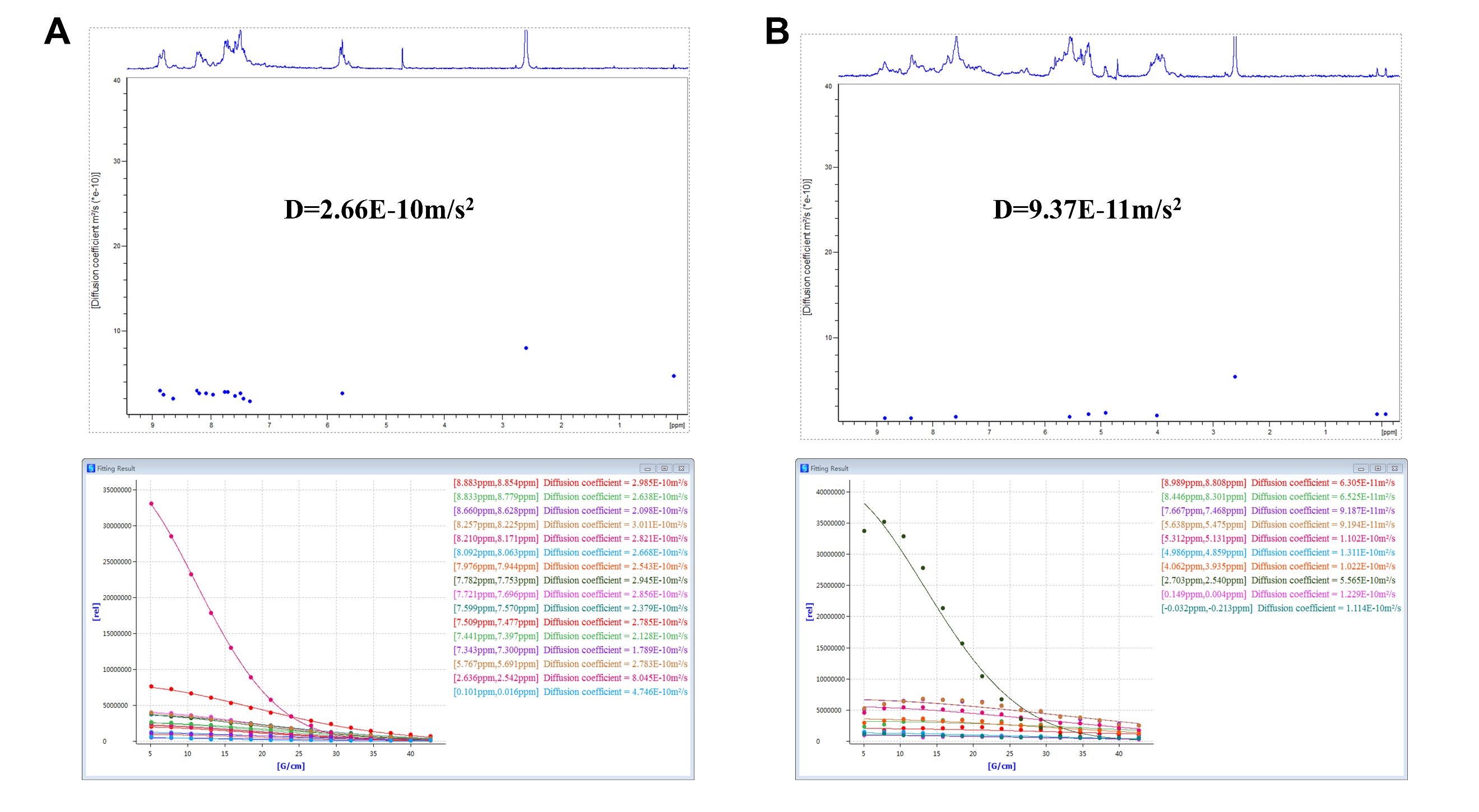


**Figure S10**. 2D DOSY spectra of **G1** and **G1**/CB[8] (400 MHz, D_2_O, 298 K).


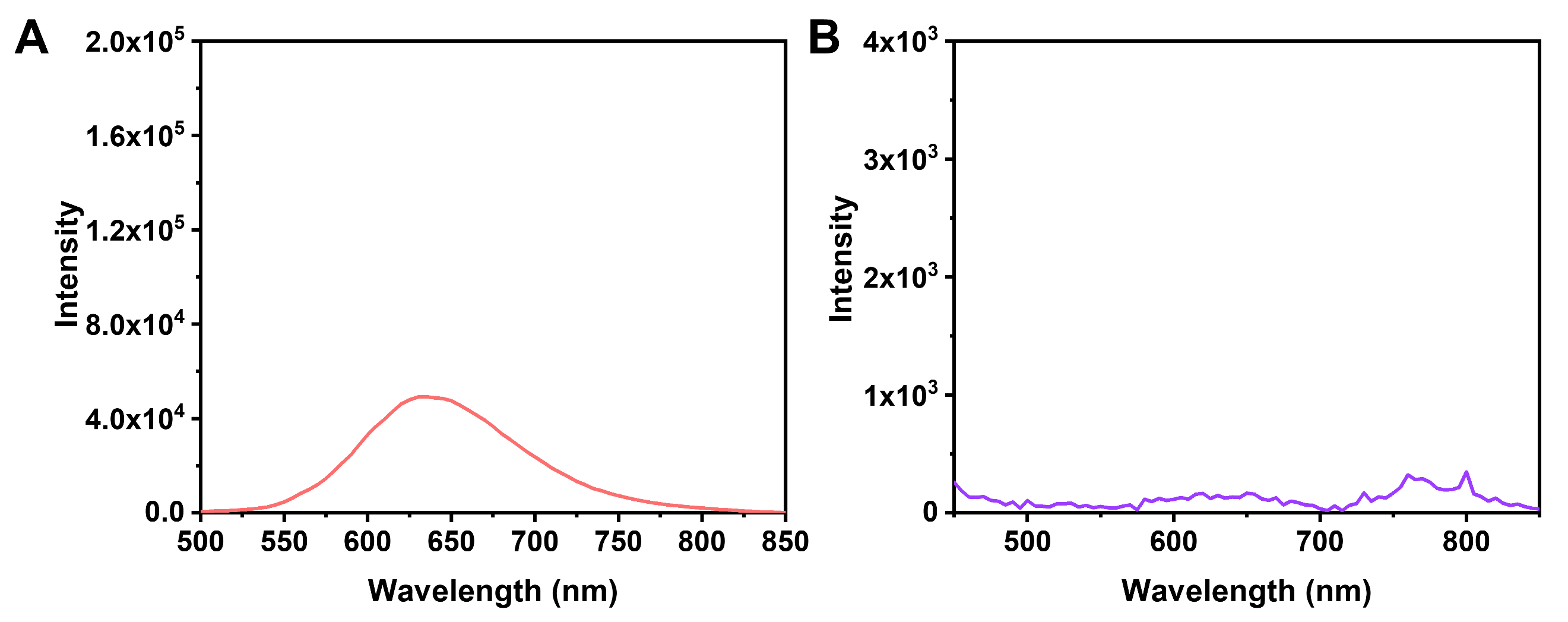


**Figure S11**. (A) The PL spectrum (λ_ex_ = 470 nm) and (B) delayed spectrum (λ_ex_ = 330 nm) of **G1** ([**G1**] = 25 μM).


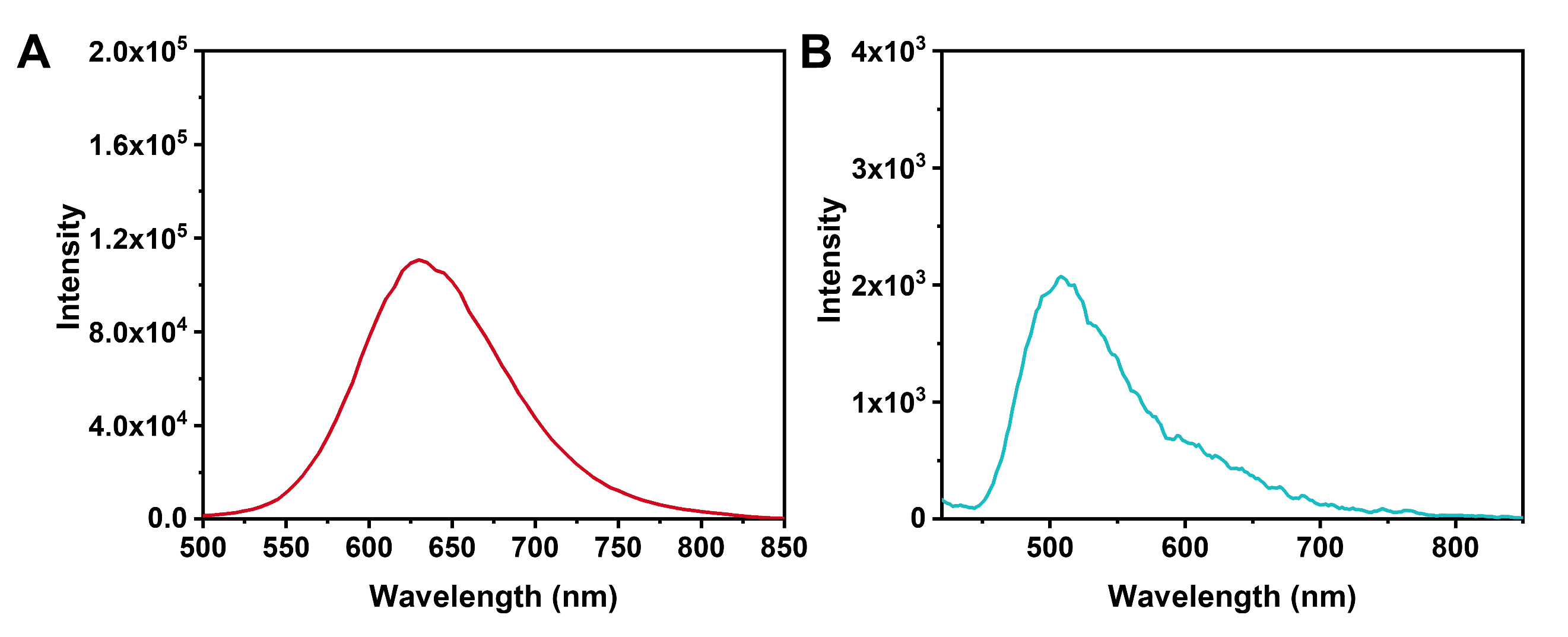


**Figure S12**. (A) The PL spectrum (λ_ex_ = 470 nm) and (B) delayed spectrum (λ_ex_ = 330 nm)

of **G1/** CB[8] ([**G1**] = [CB[8]] = 25 μM).


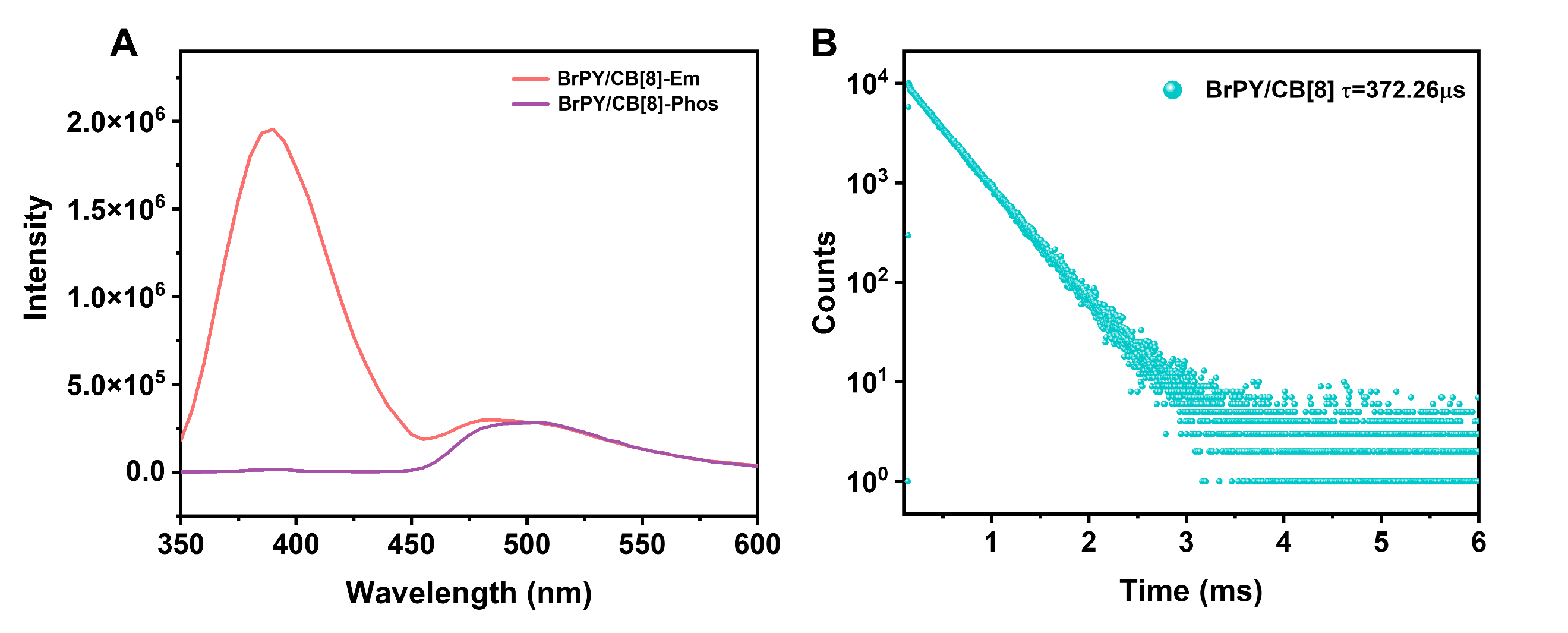


**Figure S13**. (A) The steady-state and delayed spectrum (λ_ex_ = 330 nm) of **BrPY**/CB[8]. (B) The time-correlated decay curves of **BrPY** at 500 nm (λ_ex_ = 330 nm).


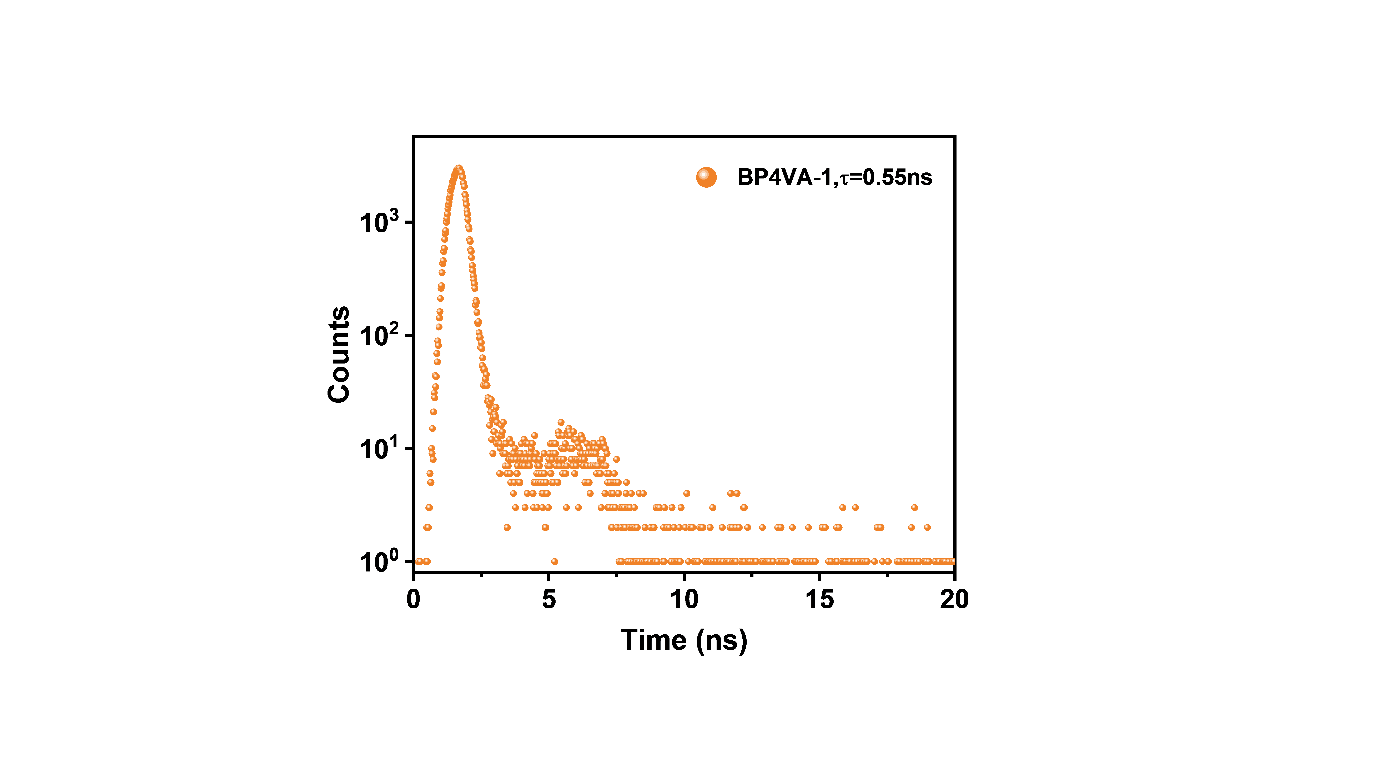


**Figure S14**. The time-correlated decay curves of **BP4VA-1** at 650 nm (λ_ex_ = 470 nm).


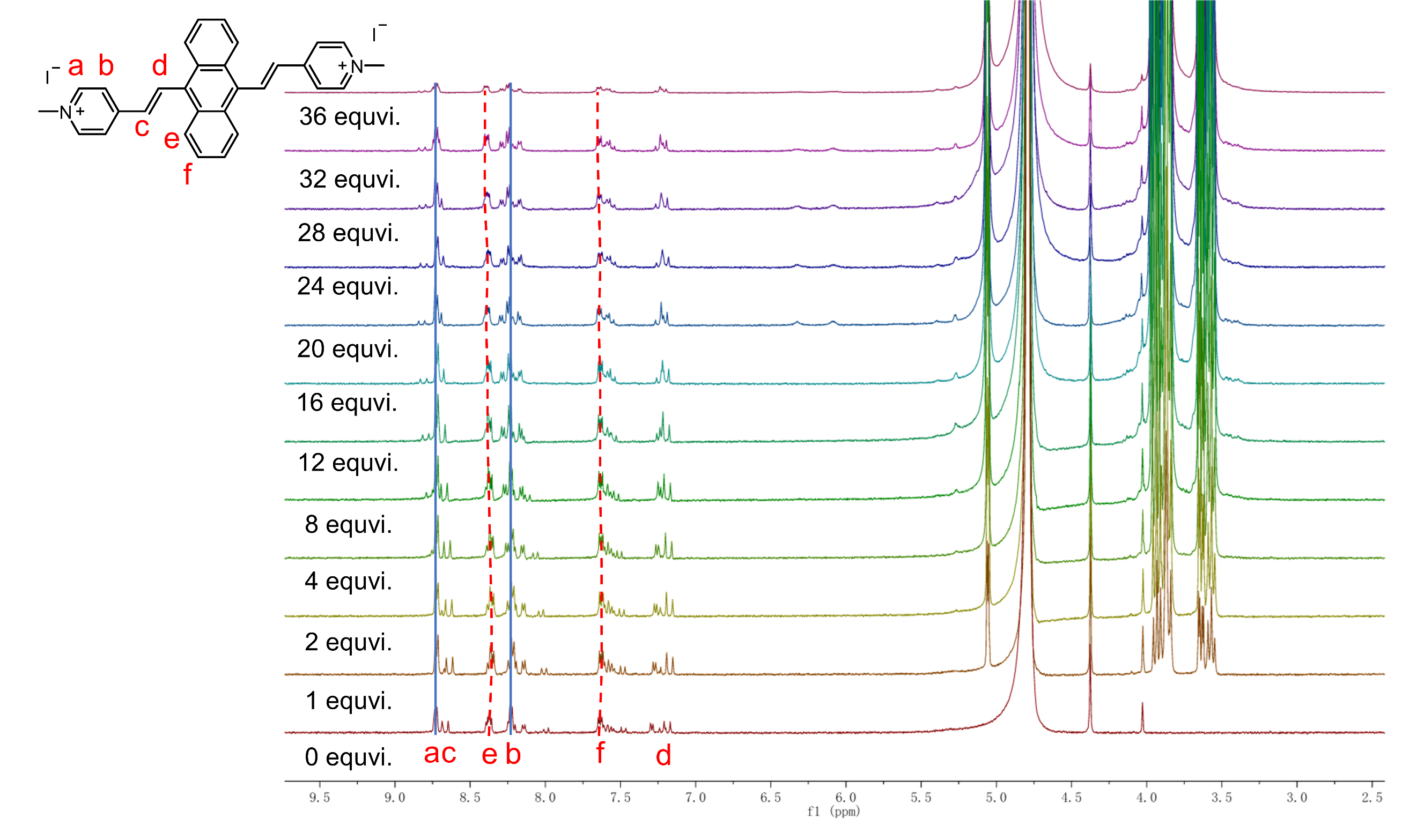


**Figure S15**. ^1^H NMR spectral changes of **BP4VA-1** after adding 0, 1, 2, 4, 8, 12, 16, 20, 24, 28, 32, 36 equivalent *β*-CD. ([**BP4VA-1**] = 1 mM, 400 MHz, D_2_O, 298 K).


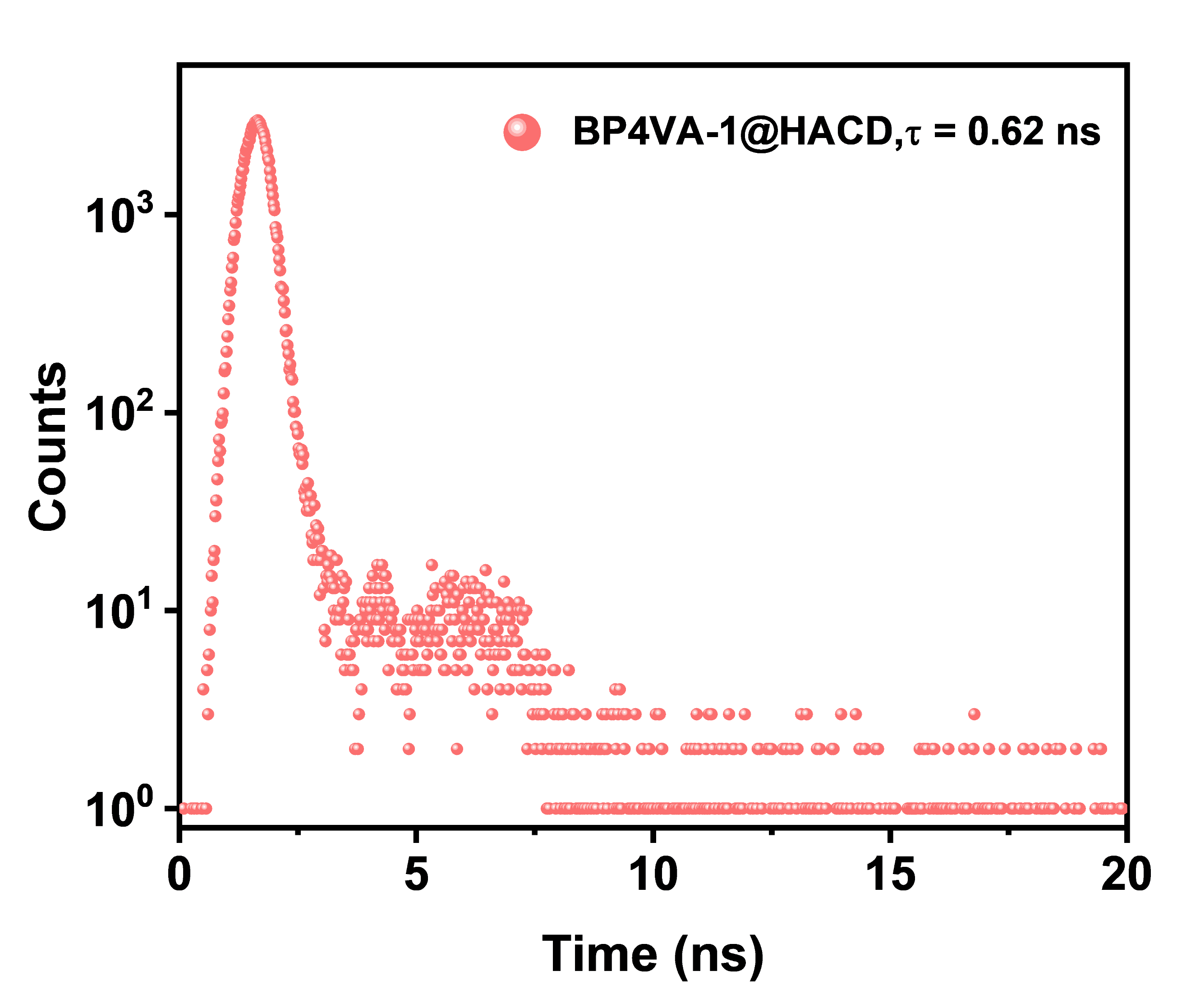


**Figure S16**. The time-correlated decay curves of **BP4VA-1**@HACD at 650 nm (λ_ex_ = 470 nm).


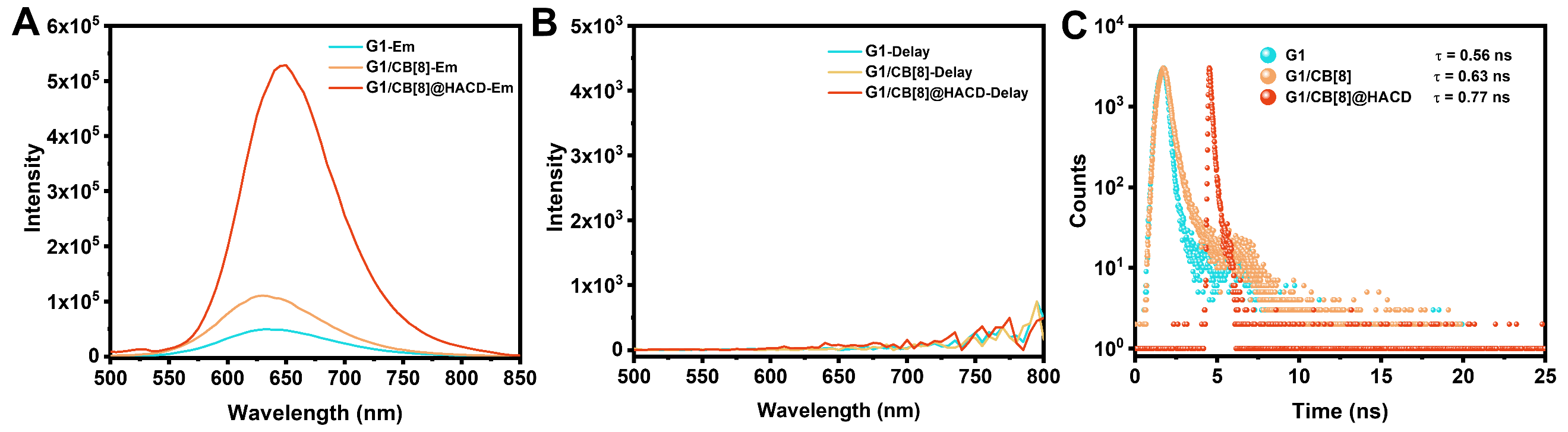


**Figure S17**. (A) The PL spectrum, (B) delayed spectrum, and (C) time-correlated decay curves at 650 nm of **G1**, **G1**/CB[8], and **G1**/CB[8]@HACD (λ_ex_ = 470 nm, [**G1**] = 25 μM, [CB[8]] = 25 μM, [HACD] = 0.045 mg/ml).


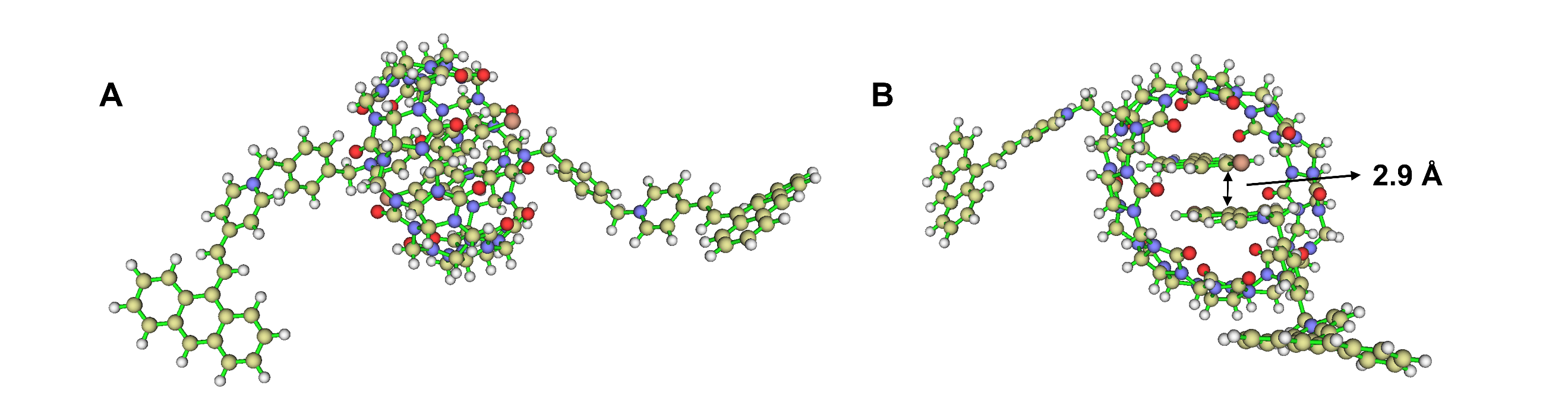


**Figure S18**. Optimized ground-state geometry of the **G1/**CB[8]. (A) Top view and (B) side view of the geometry-optimized structure, highlighting the confined arrangement of the dimer inside the CB[8] cavity. The short intermolecular contact distance (~2.9 Å) suggests strong host–guest interactions and close packing induced by CB[8] encapsulation.


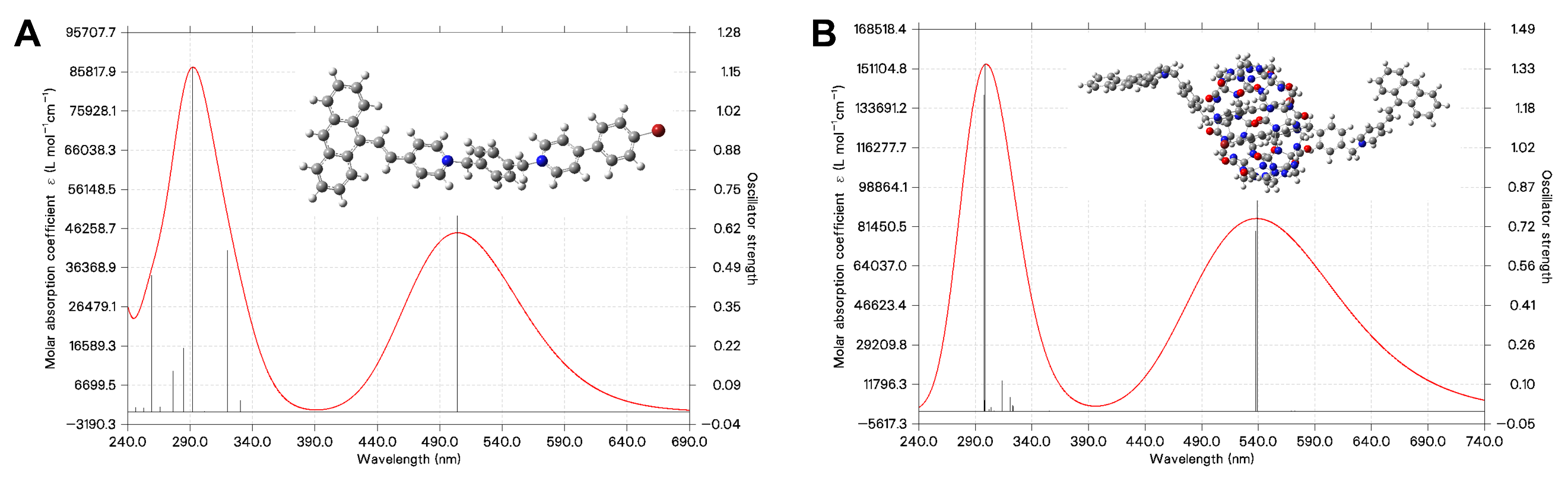


**Figure S19**. The Calculated UV–vis absorption spectra of (A) **G1** and (B) **G1/**CB[8] obtained from TD-DFT calculations. The red curves represent the simulated absorption spectra after spectral broadening, while the gray vertical lines indicate the oscillator strengths of individual electronic transitions.


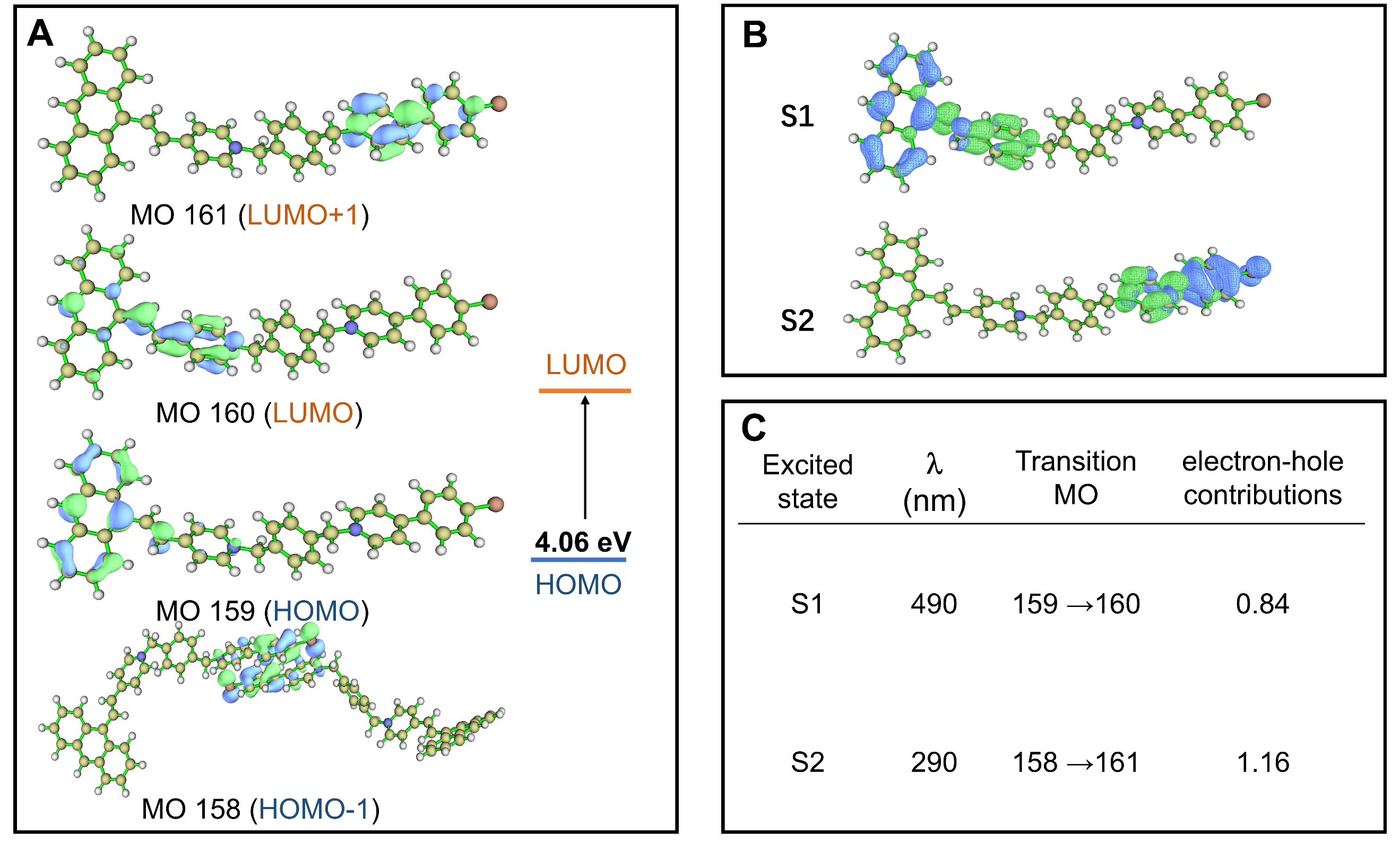


**Figure S20**. Frontier molecular orbitals and excited-state electronic characteristics of **G1** obtained from DFT/TD-DFT calculations. (A) Selected frontier molecular orbitals (HOMO−1, HOMO, LUMO and LUMO+1) with the corresponding HOMO–LUMO energy gap. (B) Electron–hole distribution analyses for the S1 and S2 excited states. (C) Summary of the dominant electronic transitions, excitation wavelengths, and electron–hole contributions for the relevant excited states.


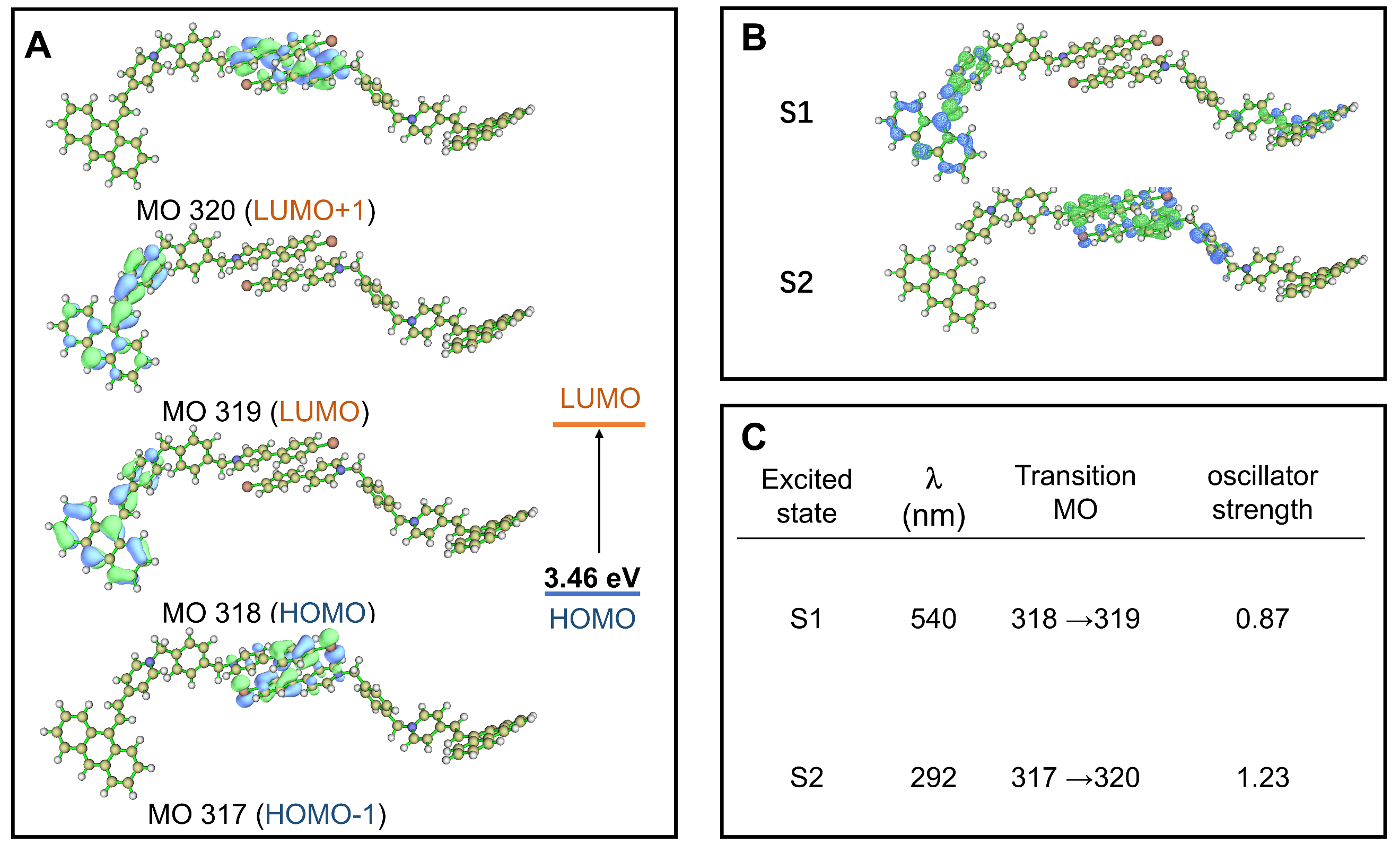


**Figure S21**. DFT/TD-DFT analysis of **G1/**CB[8]. (A) Selected frontier molecular orbitals (HOMO−1, HOMO, LUMO and LUMO+1) and the corresponding HOMO–LUMO energy gap. (B) Electron–hole distribution analyses for the S1 and S2 excited states. (C) Summary of the dominant electronic transitions, excitation wavelengths, and oscillator strengths. For clarity, the CB[8] is omitted in Figure S21A and S21B for better visualization.


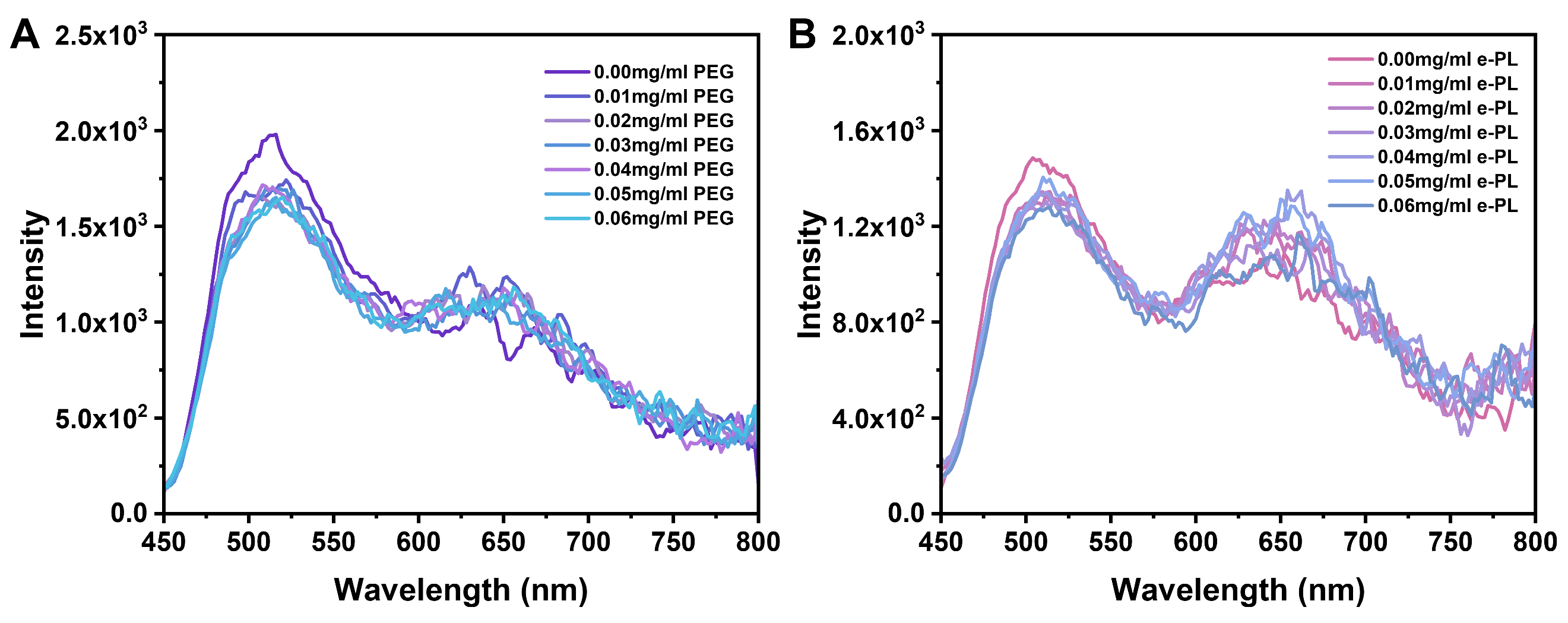


**Figure S22**. The Phosphorescence spectra of **G1**/CB[8] upon the addition of 0-0.06 mg/ml (A) PEG and (B) ε-PL ([**G1**] = [CB[8]] = 25 μM).


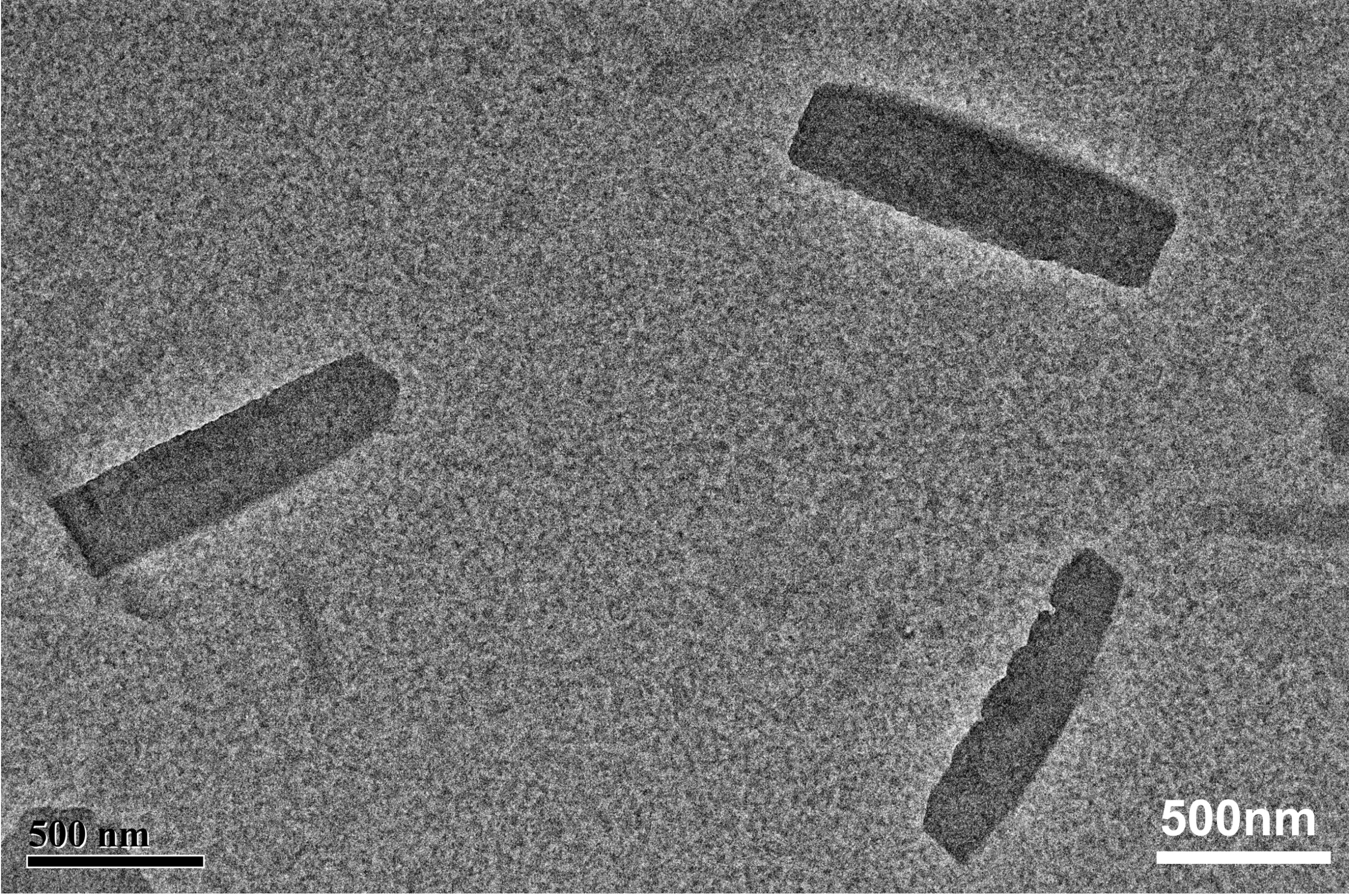


**Figure S23**. TEM image of **G1**/CB[8]@HACD upon degradation by hyaluronidase.


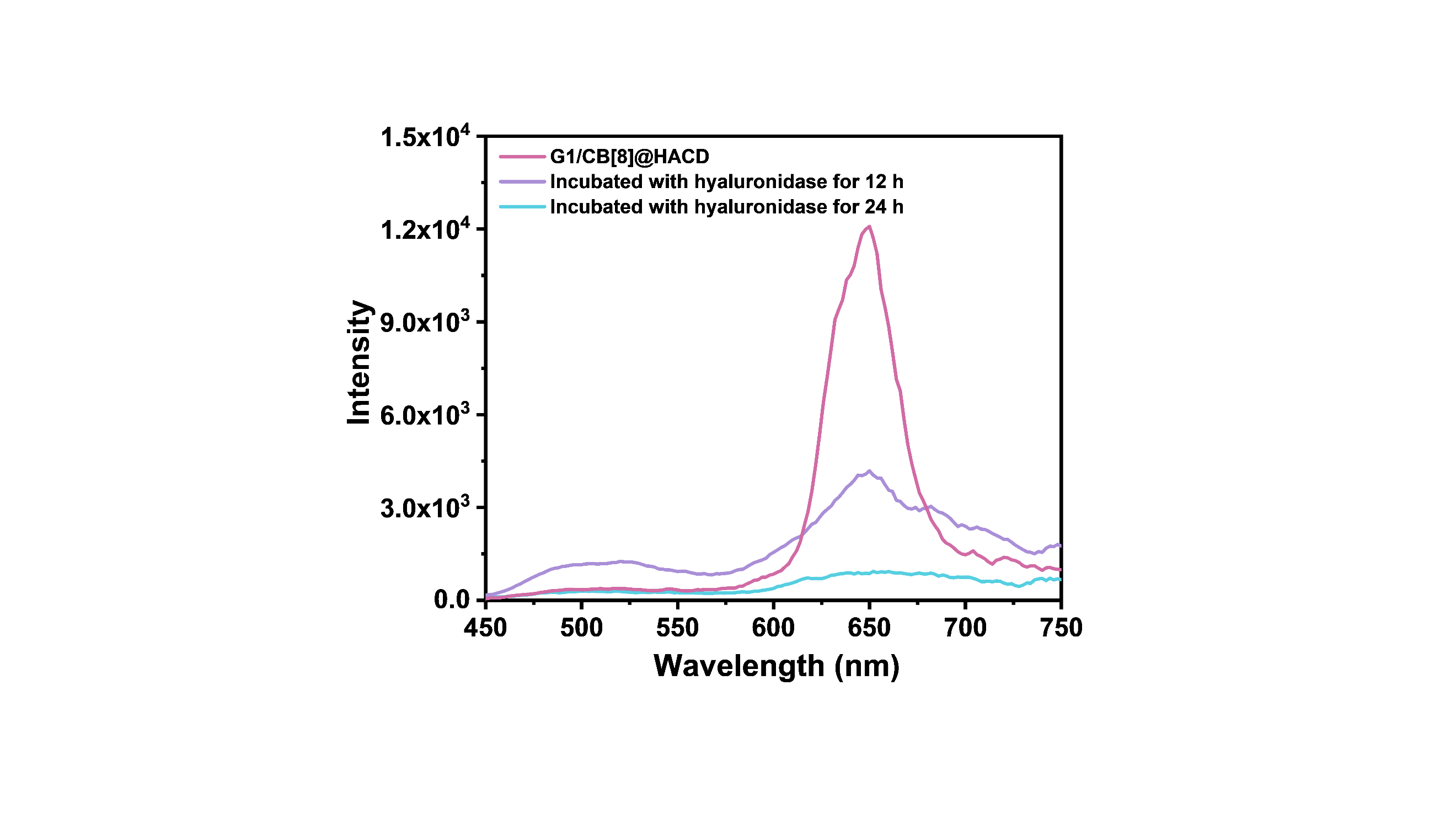


**Figure S24.** Phosphorescence spectra of **G1**/CB[8]@HACD upon the addition of hyaluronidase and incubated at 37 ℃ for 12 h and 24 h. ([**G1**] = [CB[8]] = 25 μM, [HACD] = 0.045 mg/mL, [hyaluronidase] = 0.02 mg/mL).


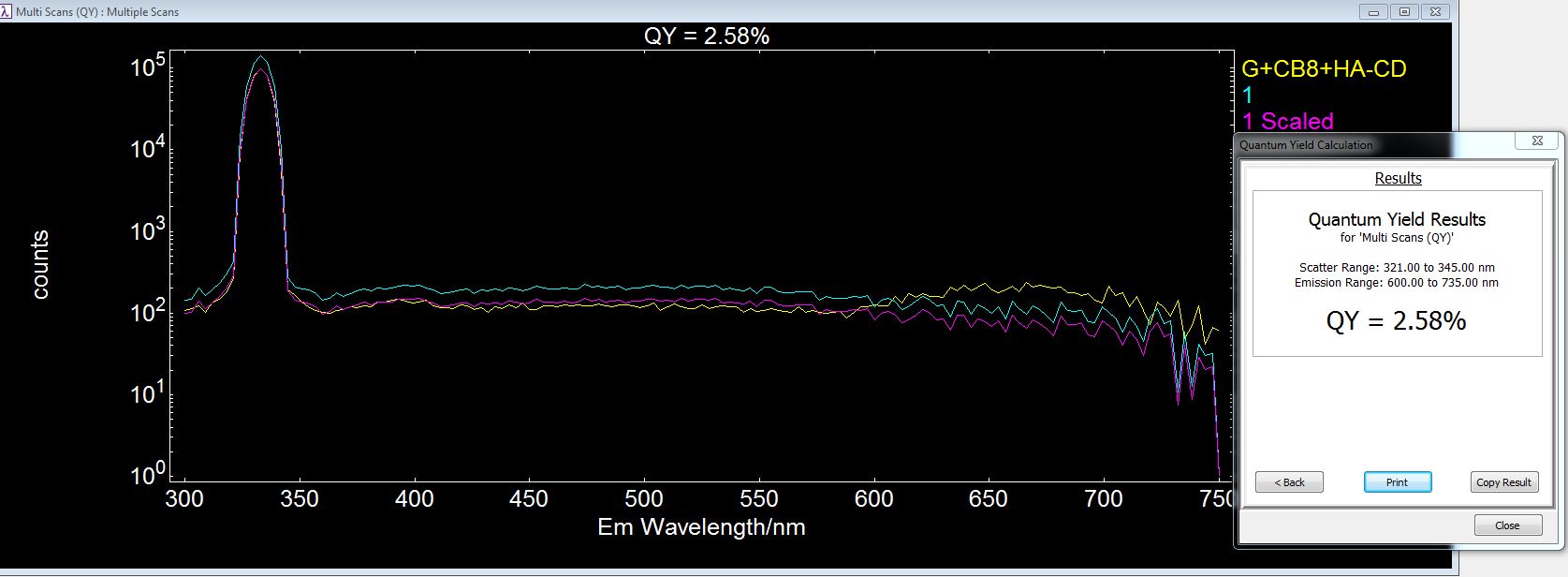


**Figure S25.** Quantum yield of **G1**/CB[8]@HACD in H_2_O. ([**G1**] = [CB[8]] = 25 μM, [HACD] = 0.045 mg/mL).


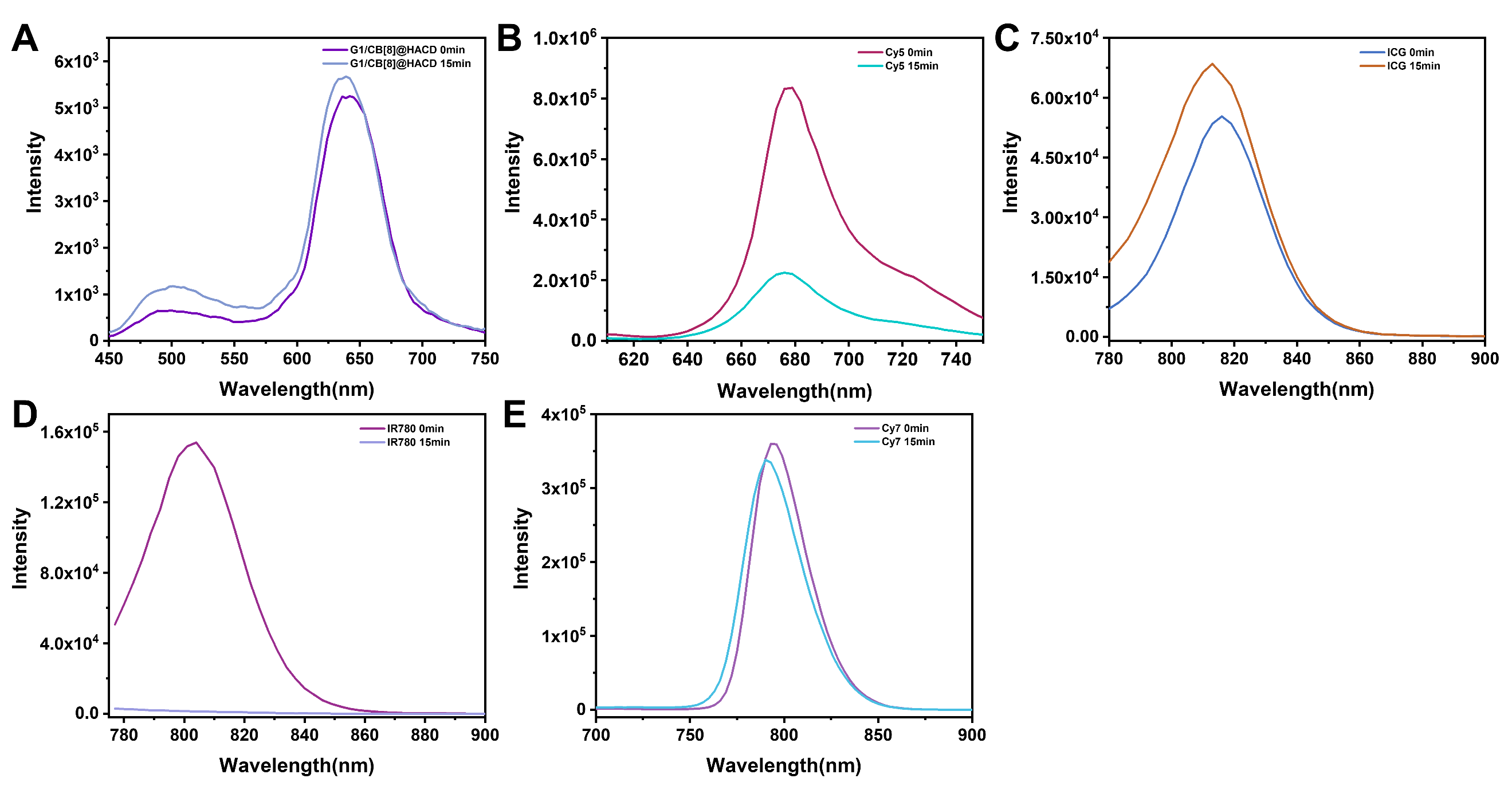


**Figure S26.** Photostability tests of (A) **G1**/CB[8]@HACD, (B) Cy5, (C) ICG, (D) IR780 and (E) Cy7 in H_2_O. Five NIR dyes were irradiated by white light (＞420 nm, 220 mW/cm^2^) for 15 min and the changes in emission spectra were detected. ([**G1**] = [CB[8]] = 25 μM, [HACD] = 0.045 mg/mL, [Cy5] = [ICG] = [IR780] = [Cy7] = 25 μM).


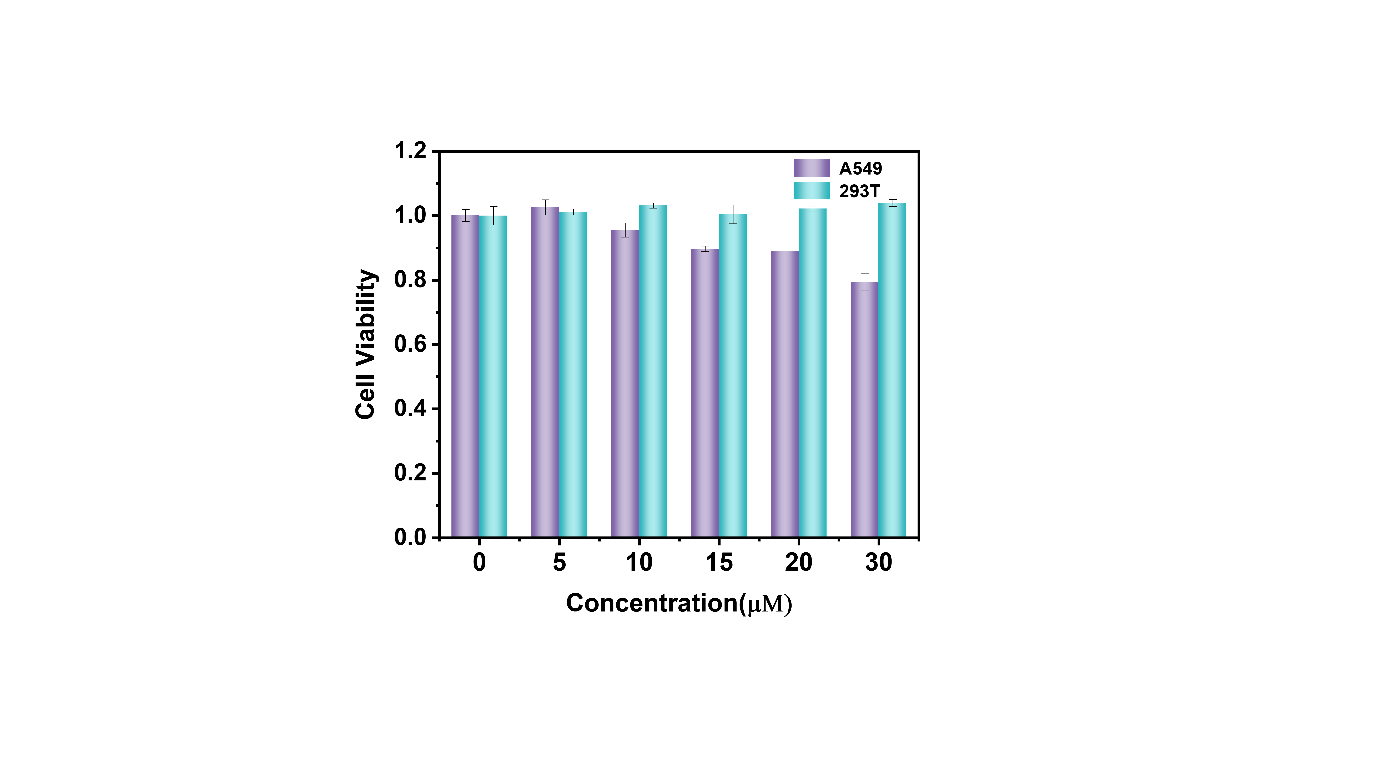


**Figure S27.** Cell viability of Hela cancer cells and 293T normal cells incubated with **G1**/CB[8]@HACD at different concentrations.

**Table S1.** The photophysical properties of the representative commercial NIR dyes and state-of-the-art PRET systems.

| NIR dyes | λ_ex_ (nm) | λ_em_ (nm) | Stokes shift (nm) | Lifetime | QY | Ref |
| --- | --- | --- | --- | --- | --- | --- |
| Cy5 | 590 | 680 | 90 | 1-2 ns | 0.2-0.25 | -- |
| Cy7 | 620 | 795 | 175 | 0.4-0.6 ns | 0.2-0.25 | -- |
| IR780 | 760 | 800 | 40 | 0.6-0.8 ns | 0.02-0.03 | -- |
| ICG | 760 | 815 | 55 | 0.2 ns | ＜0.01 | -- |
| MTPA-N | 450 | 780 | 330 | 9.1 μs | 10.74 | 3 |
| HAPY/CB[8]/G1 | 310 | 750 | 440 | 14.22 μs | 0.046 | 4 |
| G1/CB[8]/HACD/PD | 308 | 810 | 502 | 11.1 μs | 0.0267 | 5 |
| G⊂CB[7] @ HACD/TPPSS | 300 | 710 | 410 | 96.1 μs | 0.1674 | 6 |
| CQ | 450 | 710 | 260 | -- | 0.013 | 7 |
| This work (**G1**/CB[8]@HACD) | 330 | 650 | 320 | 11.2 μs | 0.0258 | -- |

**References**

1. N. Liu, Z. Chen, W. Fan, J. Su, T. Lin, S. Xiao, J. Meng, J. He, J. J. Vittal, J. Jiang, *Angew. Chem. Int. Ed.* **2022**, *61*, e202115205.
2. Q. Y. Li, Y. H. Deng, C. Cao, Y. X. Hong, X. R. Xue, M. J. Zhang, Y. Ge, B. F. Abrahams, J. P. Lang, *Angew. Chem. Int. Ed.* **2023**, *62*, e202306719.
3. Q. Dang, Y. Jiang, J. Wang, J. Wang, Q. Zhang, M. Zhang, S. Luo, Y. Xie, K. Pu, Q. Li, Z. Li, *Adv. Mater.* **2020**, *32*, 2006752.
4. J. Yu, H. Yu, J. Niu, Z. Lei, Y. Liu, *Nano Lett*. **2024**, *24*, 16124-16131.
5. J. Yu, J. Niu, J. Yue, L.-H. Wang, Y. Liu, *ACS Nano*, **2023**, *17*, 19349-19358.
6. X.-Y. Dai, M. Huo, X. Dong, Y.-Y. Hu, Y. Liu, *Adv. Mater*. **2022**, *34*, 2203534.
7. F.-T. Liu, S. Wang, Y.-P. Wang, P.-F. Jiang, J.-Y. Miao, B.-X. Zhao, Z.-M. Lin, *Talanta*. **2024**, *275*, 126135.
